# TFCheckpoint database update, a cross-referencing system for transcription factors from human, mouse and rat

**DOI:** 10.1093/nar/gkad1030

**Published:** 2023-11-22

**Authors:** Marcio L Acencio, Miguel Vazquez, Konika Chawla, Astrid Lægreid, Martin Kuiper

**Affiliations:** Department of Clinical and Molecular Medicine, Norwegian University of Science and Technology, Trondheim, NO-7491, Norway; Barcelona Supercomputing Center, Barcelona, 08034, Spain; Department of Clinical and Molecular Medicine, Norwegian University of Science and Technology, Trondheim, NO-7491, Norway; Bioinformatics Core Facility, St. Olavs hospital HF, Trondheim, NO-7491, Norway; Department of Clinical and Molecular Medicine, Norwegian University of Science and Technology, Trondheim, NO-7491, Norway; Department of Biology, Norwegian University of Science and Technology, Trondheim, NO-7491, Norway

## Abstract

Prior knowledge about DNA-binding transcription factors (dbTFs), transcription co-regulators (coTFs) and general transcriptional factors (GTFs) is crucial for the study and understanding of the regulation of transcription. This is reflected by the many publications and database resources describing knowledge about TFs. We previously launched the TFCheckpoint database, an integrated resource focused on human, mouse and rat dbTFs, providing users access to a comprehensive overview of these proteins. Here, we describe TFCheckpoint 2.0 (https://www.tfcheckpoint.org/index.php), comprising 13 collections of dbTFs, coTFs and GTFs. TFCheckpoint 2.0 provides an easy and versatile cross-referencing system for users to view and download collections that may otherwise be cumbersome to find, compare and retrieve.

## Graphical Abstract

**Graphical Abstract:**
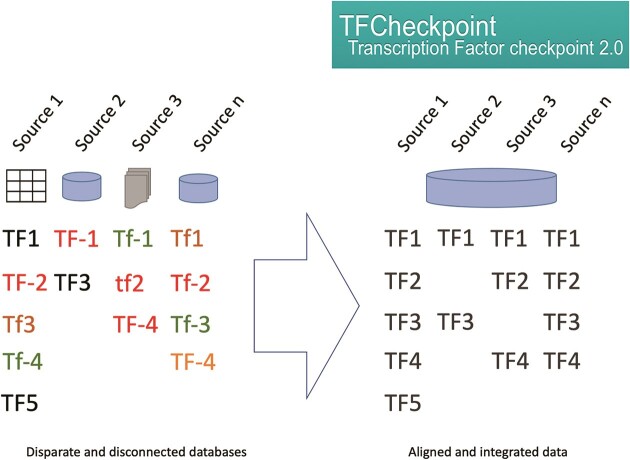

## Introduction

The regulation of transcription by RNA polymerase involves a multitude of biochemical interactions that ultimately affect the production of gene transcripts. Three types of proteins set the stage for this scenario: DNA-binding transcription factors (dbTFs), transcription co-regulators (coTFs) and general transcription factors (GTFs). In response to multiple intra- and extracellular signals, dbTFs, through their ability to bind DNA in a sequence-specific manner, guide coTFs and GTFs to specific genomic addresses where they concertedly act to finely regulate the transcription initiation burst frequency and amplitude of specific target genes. While coTFs act as bridges between dbTFs and GTFs or as chromatin modifiers that alter DNA accessibility for all types of TFs, GTFs bind promoter DNA sequences and promoter DNA-bound proteins and recruit the RNA polymerase to form the transcription preinitiation complex (PIC) (1,2).

The regulation of transcription is a vital process in all living organisms: virtually, all cellular processes, such as development, differentiation and response to external signals are governed by transcription regulation (3). Given that, access to accurate and genome-scale knowledge concerning dbTFs, coTFs and GTFs is of key importance. Multiple resources with knowledge about mammalian TFs exist, and a database integrating these various resources enables users of TF information to draw value from the work of many experts in the field.

In 2013, we launched an integrated resource focused on human, mouse and rat TFs, the TFCheckpoint database (4), to provide users access to a comprehensive overview of these proteins. Citations of the TFCheckpoint paper indicate that TFCheckpoint and its downloadable files have often been used in bioinformatics data analysis, as an alternative for the original resources. At the time of its release, TFCheckpoint covered the TF content from AnimalTFDB (5), the DBD transcription factor database (6), the Gene Ontology Annotation (GOA) database (7), JASPAR (8), the ORFeome (9), the Ravasi dataset (10), TcoF-DB (11), TFCat (12), TFClass (13) and the Vaquerizas collection (TF Census, 14). Aiming to focus on dbTFs, TFCheckpoint did not include the coTF entries in GOA, TcoF-DB or AnimalDB. We furthermore added an indication about possible curatable dbTFs for which we had identified scientific literature, pointing to the possibility to increase GO-certified annotations of dbTFs based on experimental evidence.

Here, we describe the updated TFCheckpoint 2.0 (TFC2), aligning authoritative and widely used collections of transcription factors covering dbTFs, coTFs and GTFs. Over the years, TF collections have been built according to different criteria for identification, curation and annotation, as a result of which their content, even while claiming the same objective, is partially disjunct. In addition, nomenclature conventions have undergone updates, causing name changes of proteins and genes, making cross-referencing proteins across resources a challenging task. Moreover, if the information about a TF is not stored in a database but only available from a supplementary data file or document, cross-referencing or even becoming aware of the existence of these TF ‘annotations’ becomes a very cumbersome task indeed. TFCheckpoint alleviates these problems by compiling information from both databases and supplementary files enabled by normalizing all disparate TF names to HGNC gene symbols, allowing cross referencing of all potential TFs over all resources. In addition, TFCheckpoint aligns TFs across human, mouse and rat, via orthology. TFCheckpoint is essentially a compilation of data from a wide collection of resources, and proteins are included if they have been classified as human, mouse or rat dbTF, coTF or GTF by at least one resource.

## Data sources

Since the first version of TFCheckpoint was released in 2013, several updates of the original TF databases and also new lists and database resources with mammalian TFs details have been published, reflecting continuously improving insights and evolving classification criteria. While some of the data sources have remained unchanged, such as ORFeome supplementary data (9), DBD (6), TFCat (12), the TF Census (14) and the Ravasi collection (10), others required updates, namely JASPAR (15), TcoF-DB (16), TFClass (17) and AnimalTFDB (18) (Table 1). The new resources added, all focusing on dbTFs, are the Saeed collection (19), the Lambert&Jolma collection (20) and the GO catalogue of dbTFs (21). In addition, to improve TFCheckpoint with respect to the representation of coTFs, we have now also added all proteins annotated with GO terms specific for coTFs (GOA database accessed via QuickGO [22] on 30 May 2023) and all coTF entries from TcoF-DB v2 (16) as well as AnimalTFDB version 4.0 (18). In addition, all proteins annotated with GO molecular function terms for GTF activity have also been included.

**Table 1.** Improvements relative to the first TFCheckpoint (1.0) version include the following. (‘+’ = included, ‘-’ = not included):

Data source			Presence in TFCheckpoint	
Name	Current availability	Original/first publication	TFCheckpoint 1.0	TFCheckpoint 2.0
ORFeome	Supplementary material	(9)	+	+
JASPAR	Downloadable file from website after appropriate search strategy	(8)	JASPAR 2008	JASPAR 2022
DBD	Unavailable; carried over from TFCheckpoint 1.0	(6)	+	+
TFCat	Unavailable; carried over from TFCheckpoint 1.0	(12)	+	+
TF Census (Vaquerizas collection)	Supplementary material	(14)	+	+
Ravasi collection	Supplementary material	(10)	+	+
TcoF-DB	Downloadable files from website	(11)	TcoF-DB v1	TcoF-DB v2
AnimalTFDB	Downloadable files from website	(5)	Version 1.0	Version 4.0
TFClass	Downloadable files from website	(13)	2013 version	2018 version
Saeed collection	Supplementary material	(19)	-	+
The Human Transcription Factors (Lambert&Jolma collection)	Downloadable file from website	(20)	-	+
GO catalogue of dbTFs (Lovering collection)	Supplementary material and non-downloadable catalogue from QuickGO	(21)	-	+
dbTF, coTF, GTF annotated proteins from GO Consortium (GOC)	Downloadable file from website after appropriate search strategy	https://www.ebi.ac.uk/QuickGO/ (22)	-	2023 version

## Data collection and processing

To be part of the TFC2 collection, genes from the original sources have to meet the following criteria: (1) be classified as dbTF, coTF or GTF according to the principles of the sources, (2) be from human, mouse or rat origin and (3) be mapped to the UniProt Knowledgebase (UniProtKB) (23), to be more specific, to the manually annotated and reviewed Swiss-Prot section of UniProtKB.

Data were collected by first scanning the biomedical literature in search of updated versions of data sources already present in the original version of TFCheckpoint, and to identify new sources dedicated to cataloguing human, mouse and rat dbTFs, coTFs and GTFs. With regard to the GO database, we retrieved dbTFs, coTFs and GTFs proteins associated with the following molecular terms and children thereof: ‘DNA-binding transcription factor activity’ (GO:0003700) for dbTFs, ‘transcription coregulator activity’ (GO:0003712) for coTFs and ‘general transcription initiation factor activity’ (GO:0140223) supported by any type of evidence. These GO terms are listed in Supplementary Table S1 together with some other TF relevant GO-term annotations that we have provided for the proteins in TFCheckpoint in our database. Upon selecting the sources, we downloaded the TFs list-containing text files from the source websites or used the supplementary files accompanying the original publication (Table 1). In some sources (ORFeome, Vaquerizas, Ravasi, TcoF-DB, Animal TFDB, Saeed, Lambert&Jolma, Lovering and TFClass), the files were promptly available from download, while in others, specifically JASPAR and GO database via QuickGO, we had to provide specific queries via REST APIs to retrieve the lists of interest. Except for TFClass, all sources provided files specific for a TF type, i.e. dbTF, coTF and GTF, and organism, i.e. human, mouse and rat. Regarding TFClass, we had to use an in-house script to parse the provided turtle file (http://tfclass.bioinf.med.uni-goettingen.de/about.jsf). As the DBD and TFCat databases are currently inaccessible, we had to take these collections from TFCheckpoint 1.0.

Before proceeding to the integration of the resources, all entries were checked for updates at their sequence annotation levels and discontinued entries and entries classified as pseudogenes were removed from the resources lists. By using in-house Ruby scripts (https://github.com/Rbbt-Workflows/TFCheckpoint), these lists were individually translated from their original identifiers into gene symbols, Entrez GeneID, Ensembl and UniProt IDs and appropriately merged into one master table showing the presence and absence of TFs per data source. Entries missing any type of identifier were removed. Finally, we connected human, mouse and rat proteins by way of orthology information obtained from the OrthoDB database (v10.1) (24). In brief, we used OrthoDB’s SPARQL endpoint to obtain all human, mouse and rat orthologous genes at the mammalian level present in OrthoDB (Supplementary Table S2). We then translated all entries from their original identifiers (Entrez Gene IDs) into gene symbols, Ensembl and UniProt IDs and merged them into one master table. Finally, we discarded entries if none of their orthologs could be mapped to UniProtKB/Swiss-Prot.

## Database content

TFC2 contains 3554 entries, 3474 of which are human proteins and 80 are mouse proteins which are not accompanied by human orthologs (Supplementary Table S3). Each of 3554 proteins in TFC2 originates from one or more resources that at one point were considered by central research groups in the field to carry information pertinent to transcription regulation, meaning that these proteins were deemed to have a high likelihood of being a dbTF, coTF or GTF. Experimental evidence is recorded for 1199 proteins of the TFC2 entries (∼34% of total), of which 760, 434 and 43 proteins are classified as dbTFs, coTFs and GTFs, respectively (GO database accessed via QuickGO on 30 May 2023) (Supplementary Table S4). Transcription factors without experimental evidence are mainly substantiated by phylogenetic evidence types (796 proteins,∼22% of total). GO computational evidence is available for approximately half of all GO-annotated transcription factors, with five dbTFs, 14 coTFs and one GTF supported solely by computational automatic evidence. The remaining 1496 proteins are not annotated with TF function in GO. It is important for users to note that, for each resource, all entries were collected, regardless of the confidence level assigned to them by the resource providers. As an example of what a user may encounter, a query for the gene TRMT1 shows that it is present in TFC2 because of its mention in the Orfeome, Ravasi and Vaquerizas (TF Census) resources (Figure 6). TRMT1 codes for the tRNA (guanine(26)-N(2))-dimethyltransferase, an enzyme that demethylates a single guanine residue at position 26 of most tRNAs using S-adenosyl-L-methionine as donor of the methyl groups, and as such it seems very unlikely that this protein will at some point turn out to be associated with a role in gene transcription regulation. The user should be aware that the subset of 1496 proteins not annotated with a TF function in the GOA database should be used with great care. Even though many of these proteins may qualify as coTFs, considering their classification as such by TcoF-DB and AnimalTFDB, also a considerable number of them are unlikely to have TF function. Observing whether there is some consensus among the different resources is only a first step in checking proteins for TF function if they do not have such an annotation in the GO database (please refer to Supplementary Text for more details). Despite this uncertainty, we will use ‘Transcription factor’ or ‘TF’, to indicate the proteins in queries and results from TFCheckpoint, in all sections below.

Of the ten resources in the Table 1 that mainly contain dbTF proteins (ORFeome, Vaquerizas, Ravasi, Saeed, Lambert&Jolma, Lovering, TFClass, DBD, JASPAR and GOA database), there are five that have been developed explicitly to cover dbTFs as accurately and exhaustively as possible and are widely used as ‘defining’ resources for the full set of dbTFs: the GO database, Vaquerizas collection (TF Census), TFClass, Lambert&Jolma collection (The Human Transcription Factors), and the Lovering collection (GO Catalogue). The Supplementary Figure S1 provides cumulative citation numbers for ten of the resources and shows that many of the resources are frequently cited, including the five mentioned above.

**Figure 1.**
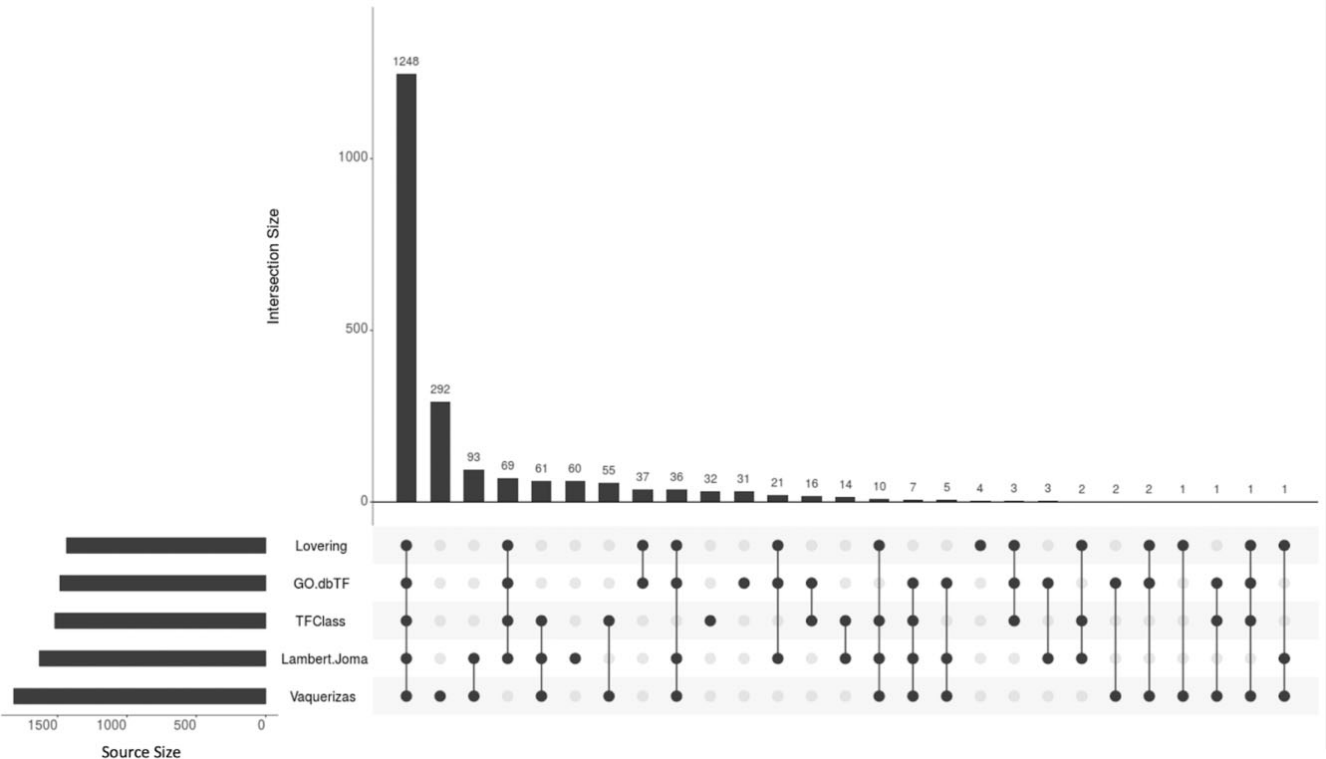
UpSet plot depicting shared and unique proteins among the five ‘defining’ dbTF sources in TFC2: the dbTF content in GO (GO.dbTF), the GO Catalogue (Lovering), the Lambert&Jolma collection (Lambert.Joma) the Vaquerizas collection (Vaquerizas) and TFClass (a total of 2106 proteins). The bars are placed in order of number of shared entries. The UpSet plot was built using the R package UpSetR, a package for the visualization of several intersecting sets (34).

The upset plot for comparing the content of the five recent and ‘defining’ dbTF resources (Figure 1) shows that the majority of dbTFs (1248) is presented by all five resources, indicating for these proteins not only a good consensus on what constitutes a dbTF but also demonstrating their proper annotation in the GOA database. However, 419 proteins appear in only one resource, indicating that today there is no absolute agreement on what should be considered a dbTF. The largest number of unique proteins (292) is present in the TF Census, of which 235 proteins are classified as ‘unlikely TF’ (TF Census classification ‘x’) with the remaining 57 entries classified as ‘probable TFs’ or ‘possible TFs’ (for details regarding TF Census classification of confidence; 14). The second largest number of unique proteins comes from the Lambert&Jolma collection (60 proteins), indicating that certainly with respect to GO annotations, this collection warrants more attention from experimentalists to produce evidence that can support GO annotators.

**Figure 2.**
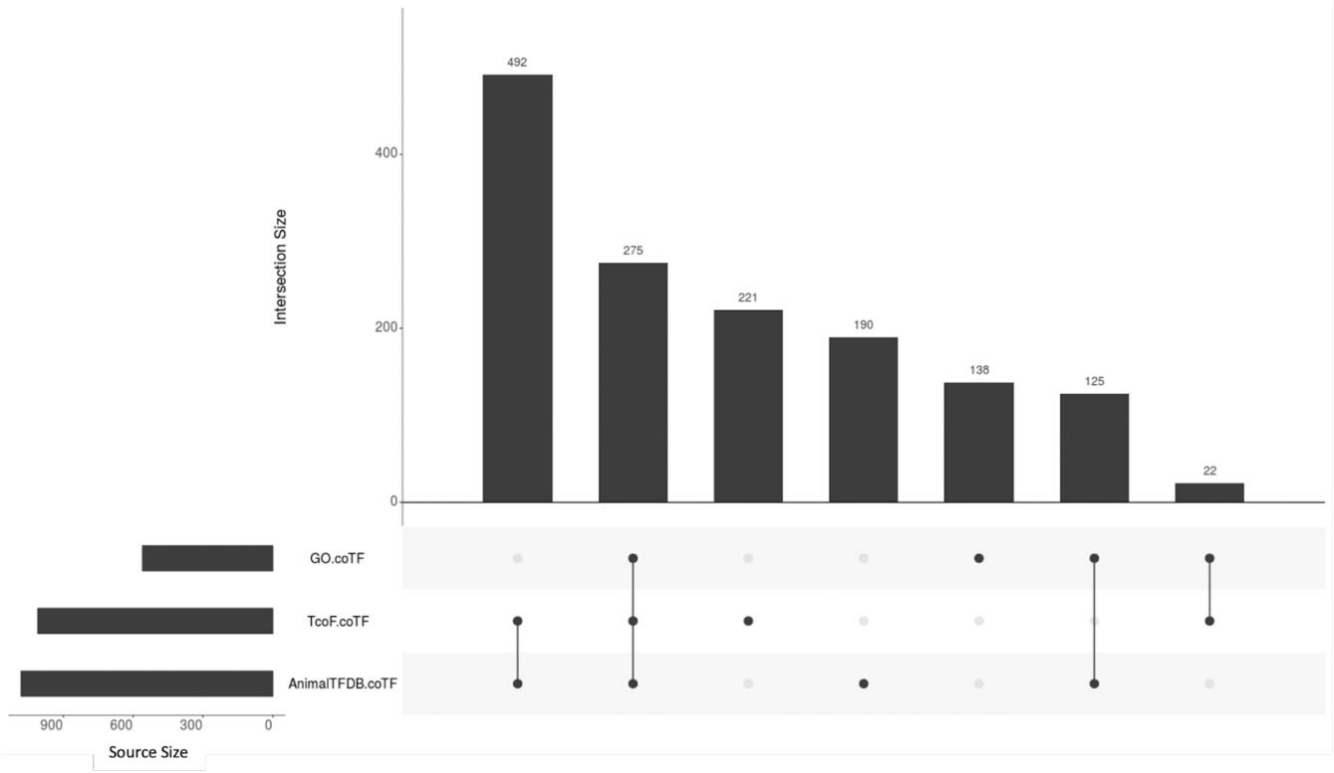
UpSet plot depicting unique and shared proteins among TFC2 sources focused on coTFs (GO_coTF, TcoF-coTF and AnimalTFDB_coTF). The bars are placed in order of the number of shared entries.

Investigation of the overlap of content of the three resources focusing coTFs, GO, TcoF-DB and AnimalTFDB (Figure 2) reveals that, contrary to the status for the five ‘defining’ dbTF resources, only a minority (275) of the 1433 proteins classified as coTF are listed in all three resources. Even between TcoF-DB and AnimalTFDB, only 492 entries are shared. Also, the percentage of proteins with GO coTF annotations among the TcoF-DB and AnimalTFDB coTF entries is much lower (19%) than was observed for the GO dbTF-annotations in the resources displayed in Figure 1. A substantial fraction of the proteins is listed in only one resource (221, 190 and 138 for TcoF-DB, AnimalTFDB and GO, respectively), suggesting that the status of coTF classification and/or their experimental study is less mature than that of dbTFs. The proteins without GO:0003712 annotations depicted in Figure 2 can be considered candidates for annotation to ‘transcription coregulator activity’ and goal oriented experimental investigations. The recently published effort by Velthuijs *et al.* (25) that similar to TcoF-DB exploits mining of protein–protein interaction databases, also represents a valuable resource for this.

The GOA database has established itself as an authoritative resource for protein function because of its rigorous annotation guidelines for protein molecular function, biological process involvement and cellular location (26,27). Active maintenance of its annotations ensures that its content provides an up-to-date assessment of protein function. We therefore have tracked all proteins annotated with GO terms that identify dbTFs or coTFs or GTFs, in the resources covered by TFCheckpoint by using a GO database download dated 30 May 2023.

**Figure 3.**
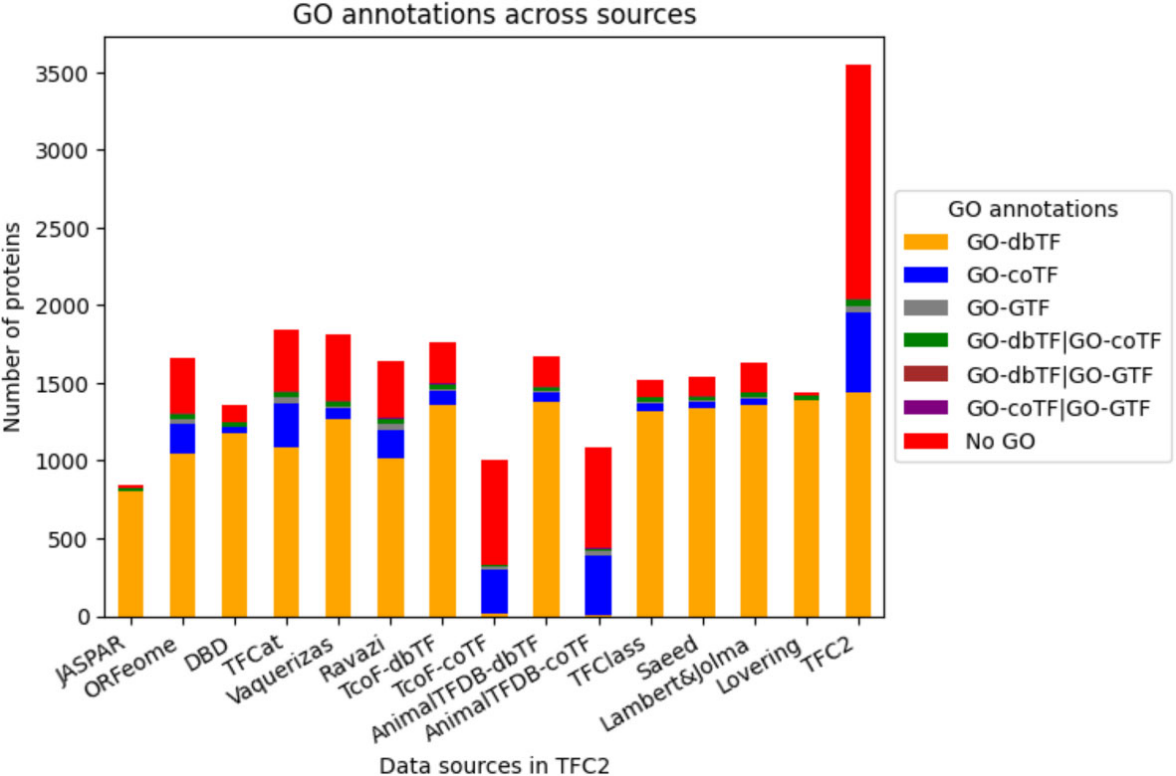
Human transcription factor GO annotation distribution across proteins listed in the TFC2 data sources. Bars present data sources, listed from left to right, according to their (first) publication year.

Figure 3 shows the coverage of each resource by GO annotations. The number of entries with dbTF GO annotations (defined by GO:0003700, coloured orange, identifying 1482 dbTF proteins in the GOA database), is consistently high across most dbTF-focused resources. The highest fraction of entries with GO dbTF annotations is found in the Lovering collection (GO catalogue) (21), which has been generated by experts active in the GO Consortium.

For the coTFs (defined by GO:0003712, coloured blue, identifying 560 human coTFs in the GOA database) the story is a bit different. In the resources dedicated to coTFs (AnimalTFDB and TcoF-DB), less than half of the entries have GO annotations for coTF function, and although some of the proteins do have annotations for GTF or dbTF, the majority of the entries have no GO annotations related to transcription factor activities (Figure 3). The 55 human proteins annotated as GTFs by GO are represented in most of the resources compiled in TFCheckpoint. In fact, GTFs are only not found in the Lovering collection (GO Catalogue). Finally, it is interesting to observe that there are also a few entries that have dual annotations, either as dbTF and coTF (39), dbTF and GTF (3), or coTF and GTF (7). These entries can be more closely inspected in Supplementary Table S5.

## Database design

The database was constructed using PostgreSQL (http://www.postgresql.org) and PHP (https://www.php.net/) was used to link the front end to the back end. The interface was designed using Joomla content management along with HTML scripting (https://www.joomla.org/). All entries are identified by their official HGNC gene symbols when human orthologs are available, or by their official MGI (for mouse) or RGD (for rat) gene symbols when human orthologs are not available (28). The HGNC, MGI and RGD gene symbols are used as the unique IDs among different tables. In addition, each entry is mapped to Entrez Gene IDs (29), Ensembl Gene IDs (30) and UniProt accessions (23) of its human, mouse and rat ortholog proteins, when available. For each entry, TFCheckpoint provides information about its presence, either directly or via any of its ortholog proteins, in the above-mentioned sources (Table 1). For the sources that allow for the construction of external links (the GOA database via QuickGO, AnimalTFDB, JASPAR, The Lambert&Jolma resource and TcoF-DB), each entry is linked out to its original source webpage. Entries classified as ‘unlikely TF’ in the original Vaquerizas collection (TF Census) are labelled with an additional ‘x’ in TFC2.

## Database usage

By using TFCheckpoint, which is accessible via the link https://tfcheckpoint.org/index.php, users can (1) obtain and download complete information for each TF, including Entrez and Ensembl Gene IDs, UniProt accessions, orthologs, transcription-related GO annotations and presence/absence in sources, (2) access the original webpage (if it exists) of sources describing the TF, (3) retrieve and download lists of TFs from one or multiple sources, and (4) download the whole content of TFCheckpoint.

**Figure 4.**
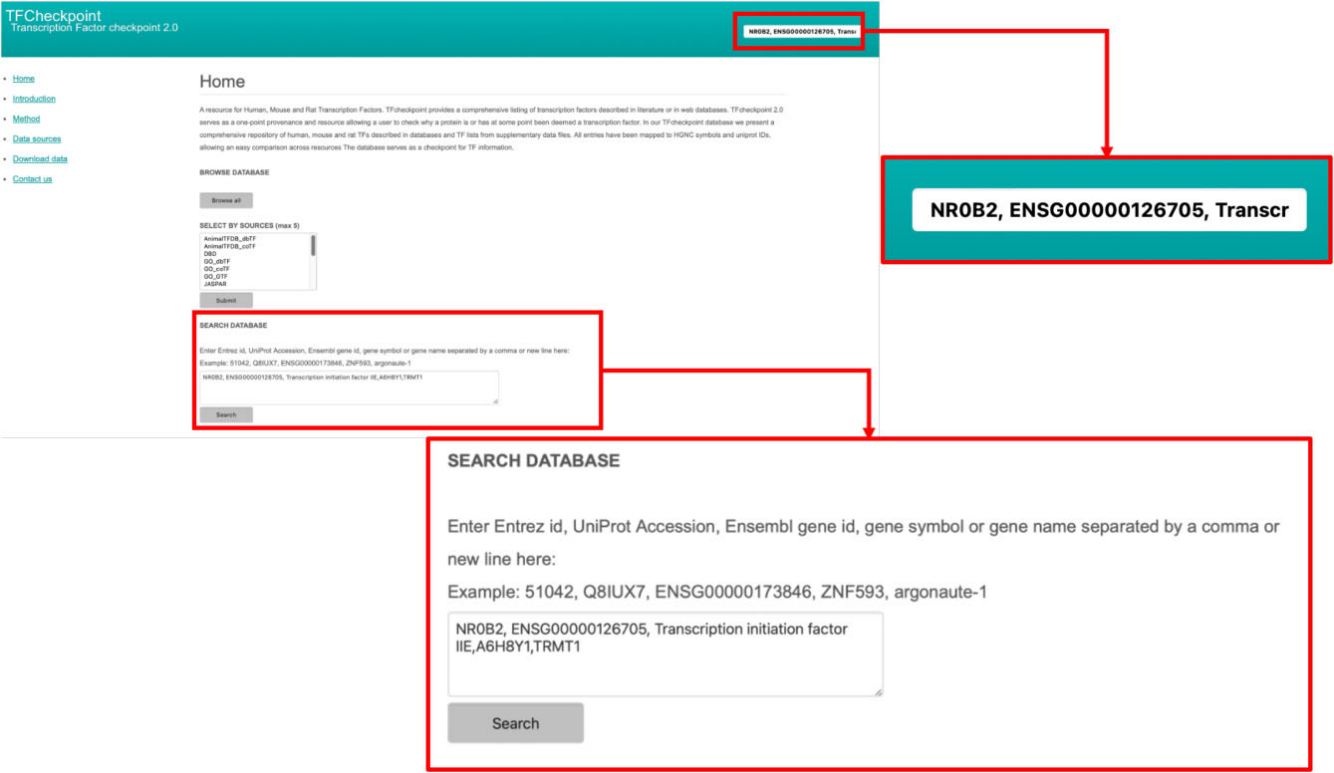
The search functions of TFCheckpoint. A user may browse all content via the ’Browse all’ button or search for one or more specific proteins identified by Entrez Gene ID, UniProt Accession, Gene name or Gene symbol through search boxes located either in the upper right of a webpage or below ‘SEARCH DATABASE’, on the homepage. As an example, the displayed query – NR0B2, ENSG00000126705, Transcription initiation factor IIE, A6H8Y1 and TRMT1 – contains proteins identified by gene symbol and name, Ensembl ID and UniProt accession.

**Figure 5.**
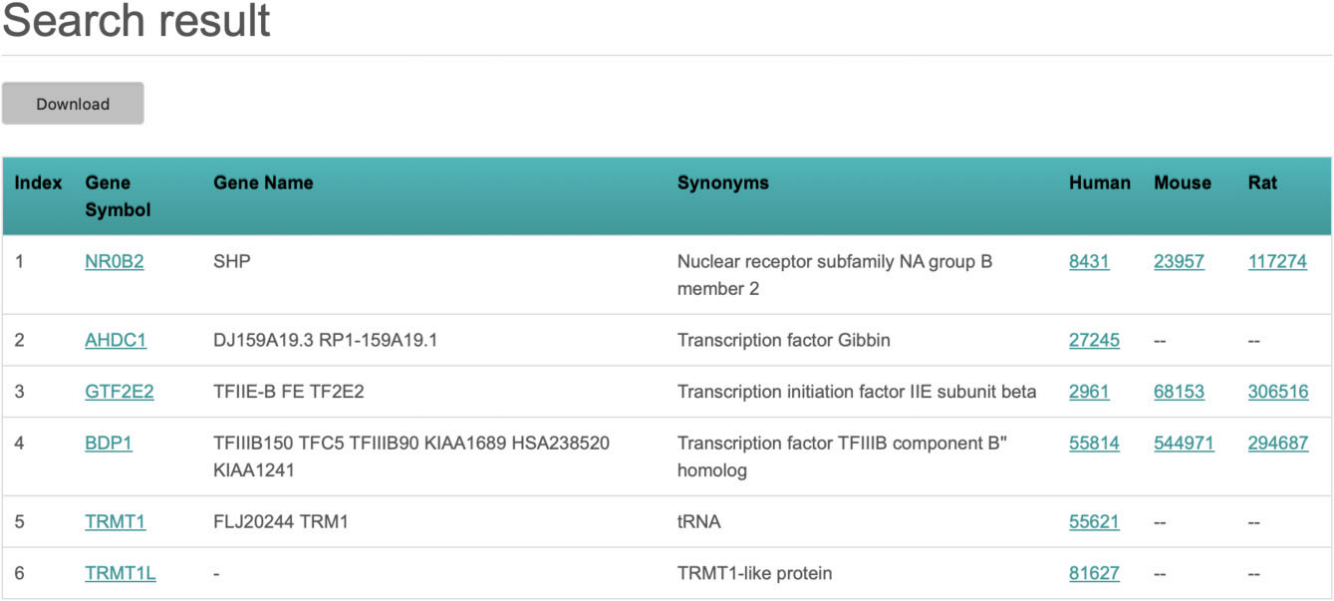
Search result page of the query ‘NR0B2, ENSG00000126705, Transcription initiation factor IIE, A6H8Y1 and TRMT1**’**. TFCheckpoint returns a list of six TFs, where TRMT1 and TRMT1L are shown because they contain the search term TRMT1 in full; the other proteins either match the Gene Symbol (NR0B2), the Gene Name (GTF2E2), the UniProt Accession (BDP1) or Ensembl ID (AHDC1). The search result page also displays synonyms and orthologs, if available.

To get information about TFs, users can either browse the whole database by clicking the button ‘Browse all’ under ‘BROWSE DATABASE’ in the homepage or input the HGNC gene name or symbol, EntrezGene ID, UniProt ID or Ensembl Gene ID of the protein(s) of interest either in the search field located in the upper right part of all TFCheckpoint web pages or in the search field in the section ‘SEARCH DATABASE’ in the TFCheckpoint homepage (Figure 4). When users would like to get information about more than one TF, commas should be used to separate entries if using the upper right search field, and commas or newlines should be used in the search field under ‘SEARCH DATABASE’ in the homepage (Figure 4). Please note that partial names or symbols are accepted as entries and, in case of searching multiple TFs, it is allowed to use different types of identifiers in the same query. By depressing the Enter or Return key after using the upper right search field or by clicking the ‘Search’ button when using the search field under ‘SEARCH DATABASE’, TFCheckpoint returns a search result page containing a list of retrieved TFs along with their HGNC symbols and names, and information about orthologs with their Entrez Gene IDs (Figure 5).

**Figure 6.**
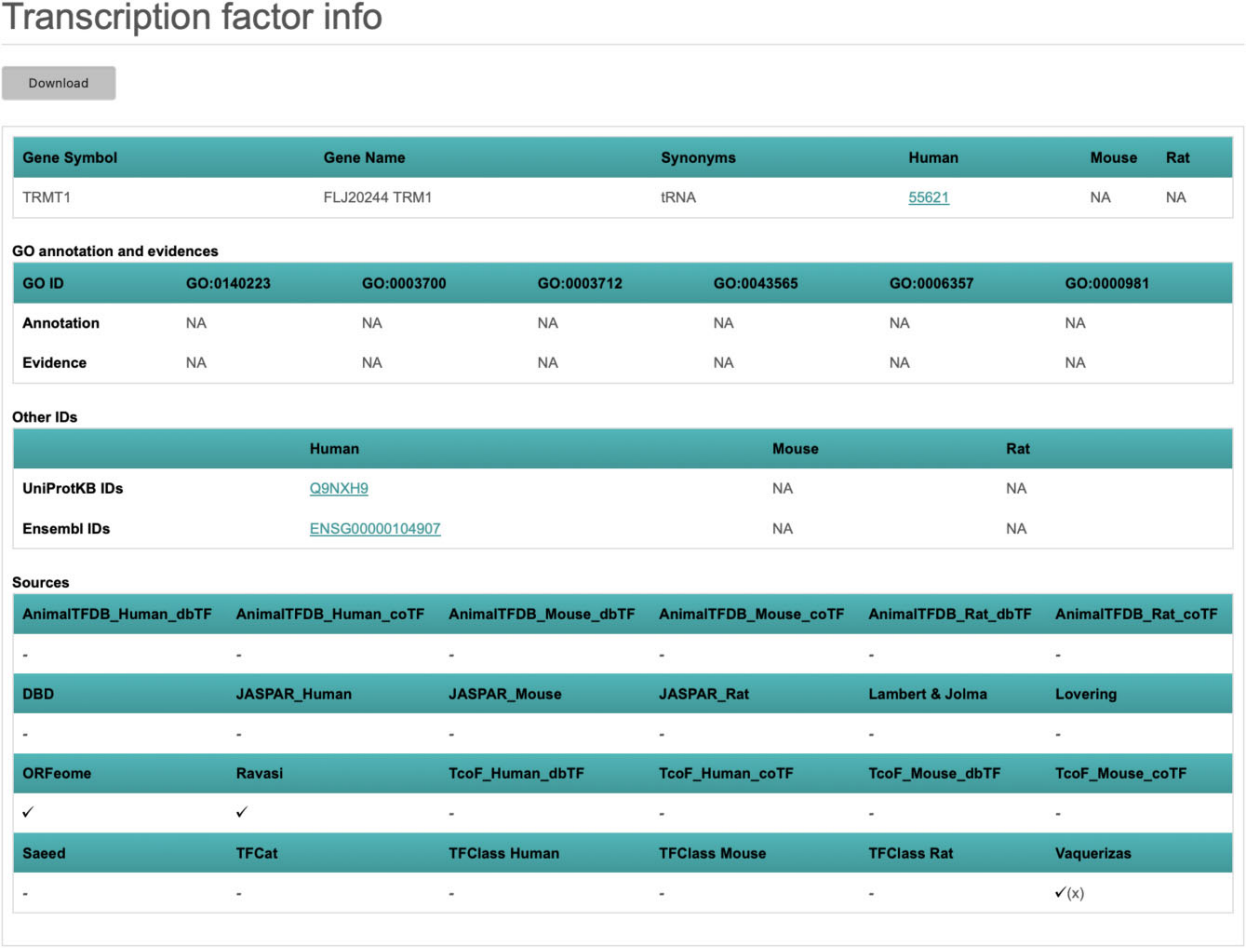
Transcription factor info page for TRMT1: in this page, users can check if the protein of interest – in this case, TRMT1 – has human, mouse and rat orthologs; in this case, no mouse or rat orthologs are available. Users can also check the GO annotation status of the protein of interest regarding transcription regulation-related GO terms; in this case,TRMT1 is not associated with any of the TFC2-selected GO annotations. In addition to official symbols and Gene IDs, other identifiers are also available for TRMT1 in this page. The presence or absence of the protein of interest in the individual TFCheckpoint sources is also shown in this page; TRMT1, for example, is listed in ORFeome, Ravasi and Vaquerizas collection of TFs. Moreover, TRMT1 is classified as an ‘unlikely TF’ in the Vaquerizas resource (14) as indicated by ‘(x)’.

**Figure 7.**
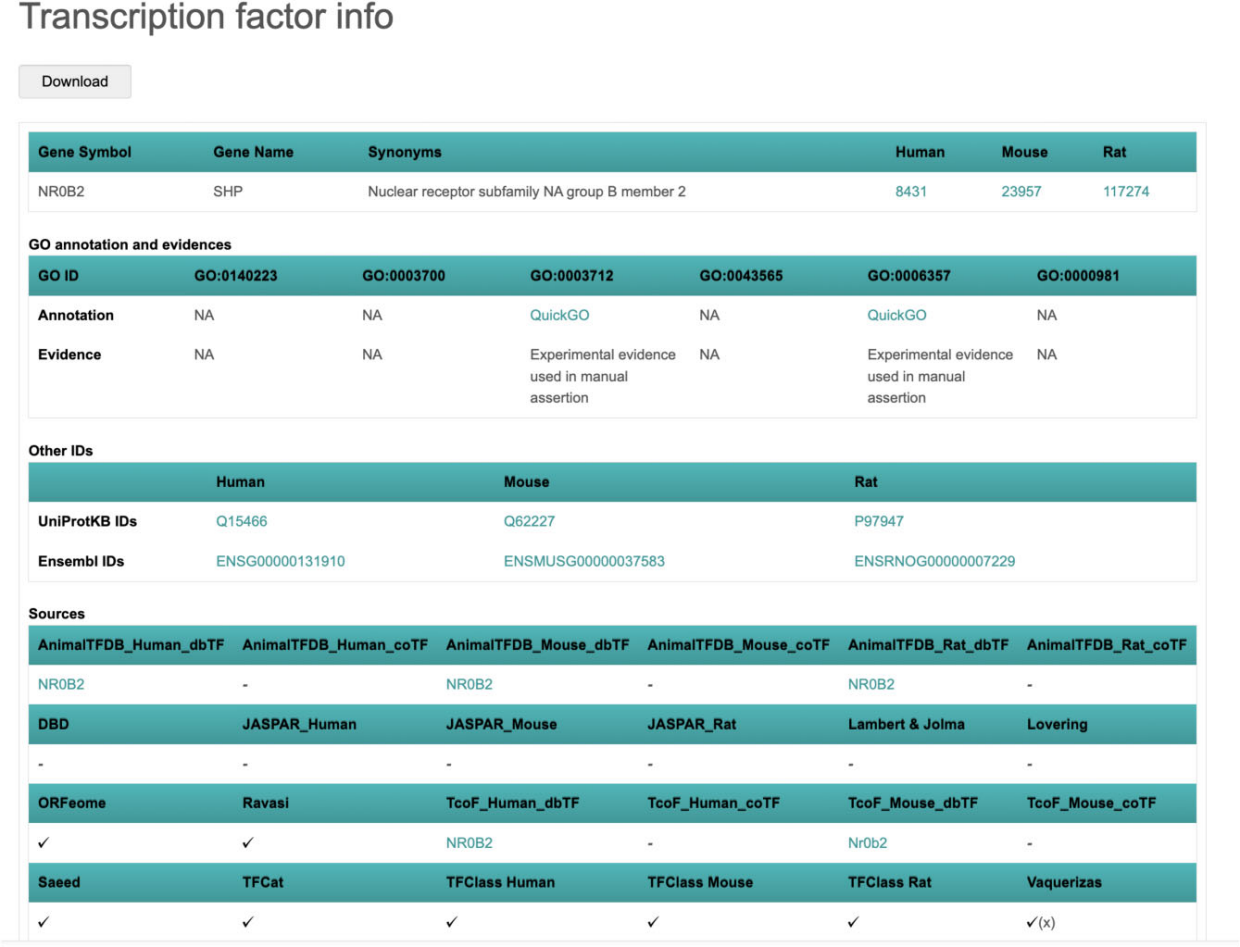
Transcription factor info page for NR0B2: in this page, users can check if the protein of interest – in this case, NR0B2 – has human, mouse and rat orthologs, which is true in this case. Users can also check the GO annotation status of the protein of interest regarding transcription regulation-related GO terms. According to GO, NR0B2 has transcription cofactor activity (GO:0003712) that is supported by experimental evidence and is involved in transcription regulation by RNA polymerase II (GO:0006357). In addition to official symbols and Gene IDs, other identifiers are also available for all NR0B2 orthologs in this page. The presence or absence of the protein of interest in the individual TFCheckpoint sources is also shown in this page; NR0B2, for example, is a member of the dbTF sets of AnimalTFDB and TcoF-DB and is listed in ORFeome, Ravasi and Vaquerizas resources of TFs; in the Vaquerizas resource, this protein is classified as ‘unlikely TF’ as indicated by ‘(x)’. When a link-out to the original source is available, the gene symbol is shown under the source name; for NR0B2, link-outs are available for AnimalTFDB and TcoF-DB.

By clicking the gene symbol of the TF of interest, TFCheckpoint returns the Transcription factor info page for that TF, containing its Entrez and Ensembl Gene IDs, UniProt accession, orthologs, transcription-related GO annotations and presence or absence in all sources integrated in TFCheckpoint (Figures 6 and 7; Supplementary Figures S3, S4 and S5). Specifically for the Vaquerizas collection (TF Census), proteins classified by the authors (14) as ‘unlikely TF’ are labelled with an additional ‘x’ (Figures 6 and 7; Supplementary S3, S4 and S5). If users want to check information about a TF in an original source, they may click the hyperlinked TF gene symbol for that source, if available (Figure 7; Supplementary S3 and S5). Finally, the results from any search action can be downloaded as a tab-delimited file, by clicking the ‘Download’ button.

**Figure 8.**
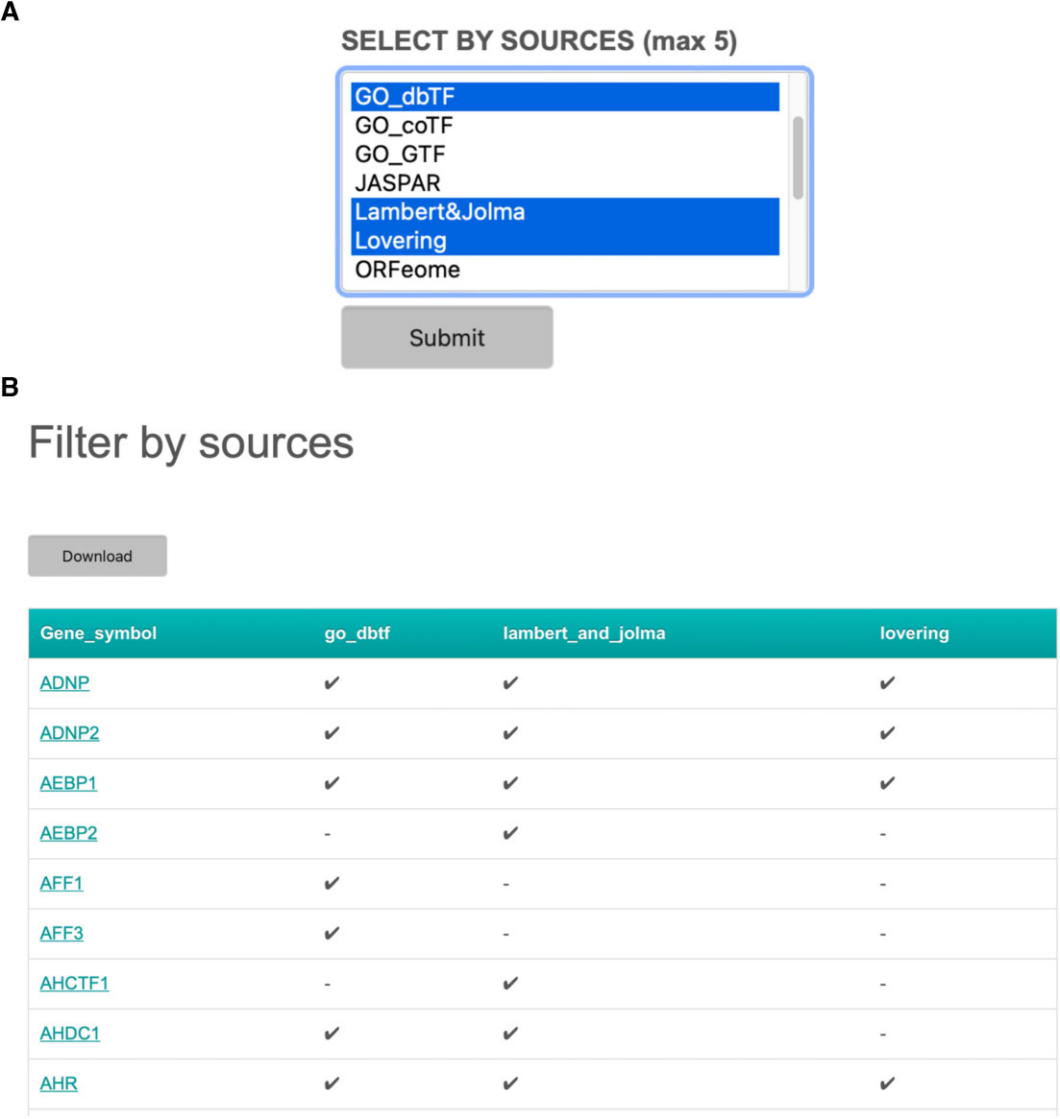
Comparing the content of several sources. (**A)** By selecting the sources (in the example: GO_dbTF, Lambert&Jolma and Lovering) in the selection box under ‘SELECT BY SOURCES’, and clicking the ‘Submit’ button, users can retrieve the aligned lists of TFs obtained from the individual sources (**B**). The results page shows, for instance, that TFs ADNP, ADNP2, AEBP1 and AHR are all present in the three selected sources, while AEBP2 and AFF3 are each present only in one source: Lambert&Jolma and GO_dbTF, respectively.

Besides obtaining information about specific TFs, users can also retrieve the TF list of one or more sources via the section ‘SELECT BY SOURCES’ on the TFCheckpoint homepage (Figure 8A). This feature is useful, for instance, if one wants to compare the content of different sources or select subsets of TFs for bioinformatics data analysis (for this specific usage, please refer to Supplementary Text). Users are allowed to select up to five sources to retrieve results. After selection, the button ‘Submit’ should be clicked upon which TFCheckpoint returns the ‘Filter by sources’ page with a table containing the content of the selected sources (Figure 8B). Proteins are identified by their HGNC gene symbols, followed by check marks that indicate their presence in a source; also here, proteins classified as ‘unlikely TF’ in the Vaquerizas resource (14) are labelled with ‘(x)’. Notice that users can also check the Transcription factor info page via this route by clicking a gene symbol. The retrieved results can be downloaded as a tab-limited file by clicking the ‘Download’ button.

Finally, the whole content of TFCheckpoint can be downloaded as an Excel or tab-delimited file, from the ‘Download data’ page of the TFCheckpoint website.

## Discussion

Comparison of the content of transcription factor resources illustrates how the definitions and classifications of these proteins have evolved over the last 15 years. Several of these resources have been recognised as authoritative in their days, only to be superseded by the next authoritative resource. The TF census (14) and TFClass (13) were the founding initiatives for genome-wide classification of proteins as dbTFs, both of them leaning heavily on bioinformatic approaches for protein structure-function classification. The Lambert&Jolma (20) and GO catalogue (21) listings of dbTFs represent the state of the art in dbTF curation aiming to substantiate bioinformatic classification with experimental and phylogenetic evidence at the individual protein level. The community efforts over the last 10–15 years to annotate the complete functional class of dbTF proteins in GO with the highest possible coverage (21,31,32) makes these functional assignments widely available to the user community, both for manual assessment of information and for computational analysis and integration through a vast number of cross-referencing systems (http://geneontology.org/docs/download-mappings/) and GO over-representation analysis dependent tools. With any resource, binary inclusion criteria (present or absent) leave room for false positives (non-dbTFs and non-coTFs that are not excluded by selection criteria that are too permissive, or inaccurate protein function knowledge) and false negatives (TFs that are not included because of strict selection criteria). Both inaccuracies pose problems for the user (33), but in particular the problem associated with proteins that experts believe are true dbTFs or coTFs but as yet lack GO annotations describing their function is underestimated. Proteins that fail to qualify for being annotated with particular functions may do so, because they have been studied less or not at all, meaning that the experimental assay data that should underpin a GO annotation is lacking. Alternatively, a publication reporting evidence on their functionality as TFs may not have been processed by GO annotators or it has been processed and it appeared to be flawed or incomplete in its evidence to make the claim (33), or there are other claims about the protein's function that do not align with what generally is accepted for a transcription factor. Access to a comprehensive collection of proteins deemed to be involved in transcription regulation by the experts that generated the various resources cited in TFCheckpoint makes it easier for users to impose their own criteria for considering further investigations, including new experiments, on proteins for which today only incomplete or disputed knowledge about possible dbTF-, coTF- or GTF-functionality is available. Decisions to look closer at proteins that are likely to be incompletely annotated with respect to their functions in transcription regulation may be taken based on various criteria: by observing that a protein has appeared in some resource that has a high standing in the community, or by judging from available GO terms and evidence codes that a protein is involved in regulation of transcription of binding of DNA (mention GO terms and evidence codes). The available PubMed ID(s) will then allow a user to perform a closer inspection.

## Supplementary Material

gkad1030_Supplemental_File

## Data Availability

TFCheckpoint 2.0 is publicly accessible without any registration or login at https://www.tfcheckpoint.org/index.php.

## Notes

Present address: Marcio Luis Acencio, Luxembourg Centre for Systems Biomedicine, Esch-sur-Alzette, Luxembourg.
