# Supplementary material for: TFCheckpoint database update, a cross-referencing system for transcription factors from human, mouse and rat
Source: Nucleic Acids Res. 2023 Nov 22;52(D1):D334–44. doi: 10.1093/nar/gkad1030 (PMC10767992; doi:10.1093/nar/gkad1030)
Supplement: gkad1030_Supplemental_File [file gkad1030_supplemental_file.pdf]

SUPPLEMENTARY MATERIAL

TFCheckpoint database update, a cross-referencing system for transcription factors from human, mouse and rat

Marcio L Acencio[1*], Miguel Vasquez[2], Konika Chawla,[1,3], Astrid Lægreid[1], Martin Kuiper[4]

1: Department of Clinical and Molecular Medicine, Norwegian University of Science and Technology, Trondheim, Norway.
2: Barcelona Supercomputing Center, Barcelona, Spain
3: Bioinformatics Core Facility, St. Olavs hospital HF, Trondheim, Norway
4: Department of Biology, Norwegian University of Science and Technology, Trondheim, Norway.

**Current affiliation: Luxembourg Centre for Systems Biomedicine, University of Luxembourg, Esch-sur-Alzette, Luxembourg.*

The Supplementary Material file contains text, figures and tables referred to in the main manuscript.

SUPPLEMENTARY TEXT

Brief guidelines on selection of TF subsets for bioinformatic data analysis

The utilization of published lists of transcription factors (TFs) has been popular among bioinformaticians that usually aim to identify TFs among long lists of genes or proteins that appear to be important in a particular context. In this regard, TFCheckpoint 2.0 (TFC2) can be very useful as several TFs from 13 disparate resources are integrated into one single and user-friendly central database. So, by using TFC2, users have a multitude of choices when the aim is to select a “ground truth” list of TFs for their specific purposes. Here we provide some quick hints on how to use TFCheckpoint to select specific DNA binding transcription factor (dbTF) and co-transcription factor (coTF) subsets for bioinformatic data analysis. We would like to emphasize fact that these quick hints are only suggestions and users are free to select subsets according to their own background knowledge:

1. dbTFs
1.1. For a comprehensive but stringent list of dbTFs: users are suggested to select only dbTFs from the Lovering collection (select “Lovering” in the “SELECT BY SOURCES” box in TFC2 homepage).

1.2. For a more comprehensive but still stringent list of dbTFs: users are suggested to select dbTFs associated with Gene Ontology (GO) annotations and those from the Lovering collection (select "Lovering" and "GO_dbTF" in the "SELECT BY SOURCES" box in TFC2 homepage).

1.3. For a more comprehensive list of dbTFs: users are suggested to select dbTFs from the five 'defining' dbTF sources (as described in the main text): dbTFs associated with GO annotations and those listed in Lovering, Lambert & Jolma, Vaquerizas and TFClass collections (select "GO_dbTF", "Lovering", "Lambert&Jolma", "Vaquerizas" and "TFClass" in the "SELECT BY SOURCES" box in TFC2 homepage); please note that the proteins in the Vaquerizas resource that are marked with with a "(x)" should not be included in this list.

1.4. For a permissive list of dbTFs: users are suggested to select dbTFs associated with GO annotations and those listed in Lovering, Lambert & Jolma, Vaquerizas, and TFClass collections as well as proteins mentioned in the dbTF sections of the TcoF-DB and AnimalTFDB (select "GO_dbTF", "Lovering", "Lambert&Jolma", "Vaquerizas", "TFClass", "TcoF_dbTF" and "AnimalTFDB_dbTF" in the "SELECT BY SOURCES" box in TFC2 homepage); please notice that the proteins in the Vaquerizas resource that are classified as 'unlikely TF', identified throughout the TFC2 database with a "(x)" should not be included in this list .

2. coTFs

2.1. For a stringent list of coTFs: users are suggested to select only coTFs associated with Gene Ontology (GO) annotations (select "GO_coTF" in the "SELECT BY SOURCES" box in TFC2 homepage).

2.2. For a less stringent list of coTFs:, users are suggested to select coTFs associated with GO annotations and those listed in the coTF sections of TcoF-DB and AnimalTFDB (select "GO_coTF", "TcoF_coTF" and "AnimalTFDB_coTF in the "SELECT BY SOURCES" box in TFC2 homepage).

SUPPLEMENTARY FIGURES

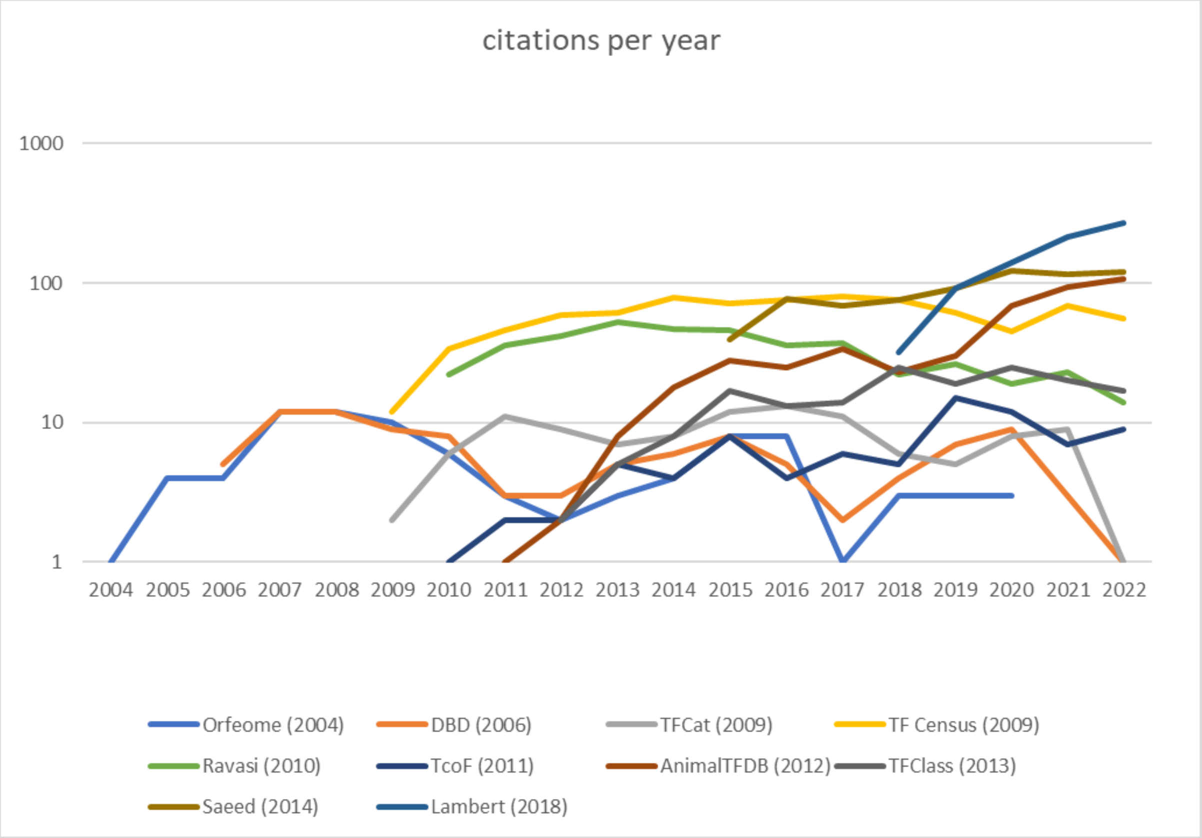

Figure S1 shows the number of citations for each resource, since their initial publication date. In case of multiple publications for the same resource, the cumulative number of citations for all publications is shown. JASPAR citations are not shown because this resource is cited for much more of its functionality than just a list of TFs. For the use of the GO database as a resource for TFs no appropriate citation number could be obtained and as for the GO Catalogue paper (Lovering et al., 2021) this paper is too recent to accumulate more than a handful of citations.

The figure has a logarithmic scale, as the number of citations varies over two orders of magnitude across resources, or even for one resource, across years. The citation numbers of most of the 'early' resources (Orfeome, DBD, TFCat and TcoF db) barely make it into the double digits, except for the TF census and the Ravasi collection, which, together with AnimalTFDB, the Saeed collection and the Lambert collection score double digit citation numbers on a yearly basis. TF census and TFClass both remain in high use. During the last years, the Lambert & Jolma collection has gained a prominent place among cited resources. The Lovering collection is still new and therefore not shown in Figure S1.

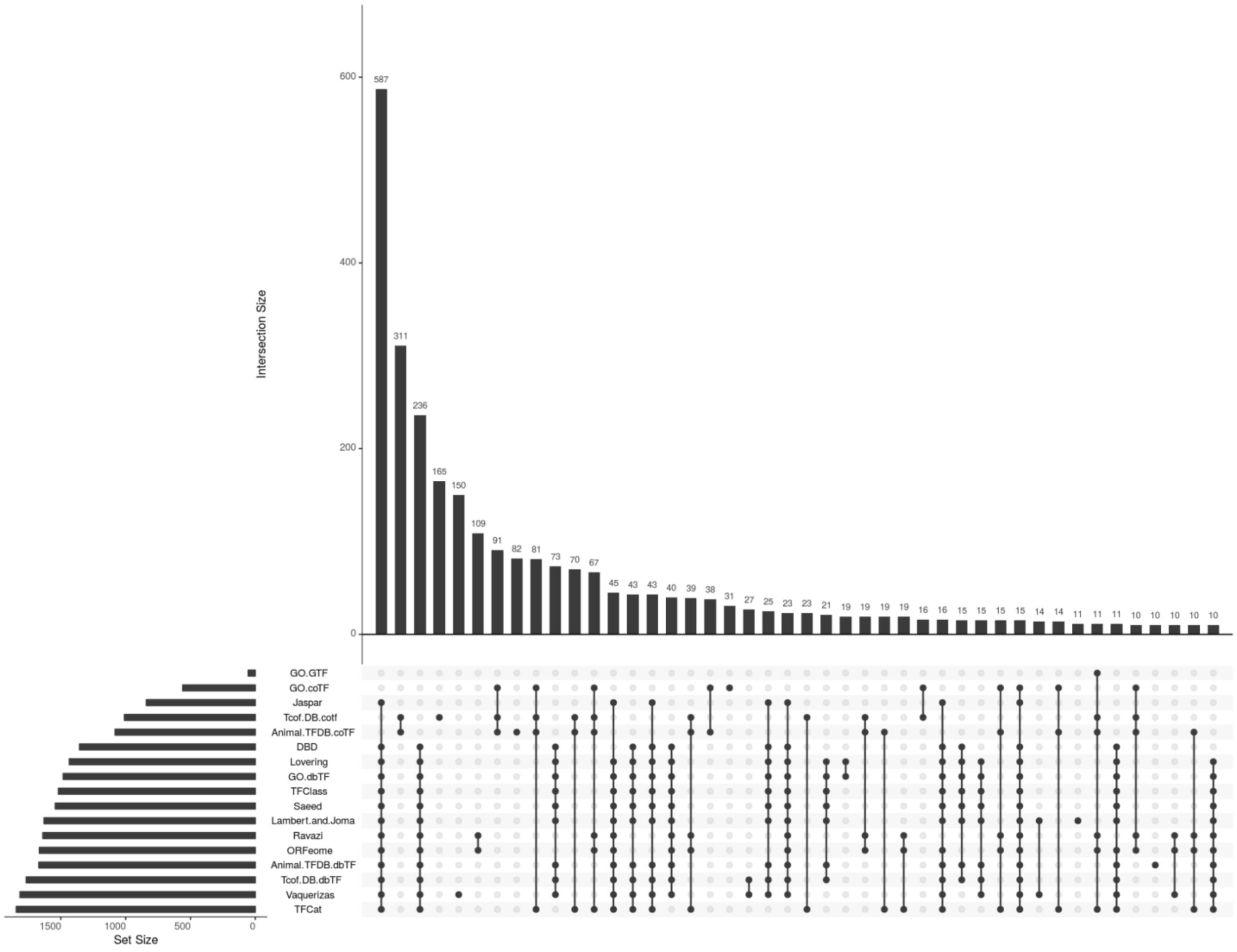

Figure S2: UpSet plot depicting shared and unique proteins among all TFC2 sources. The figure shows the 44 most frequent intersections, with a minimum of 10 proteins. The bars are placed in order of the number of shared entries. This plot shows similar trends as those depicted in Figures 1 and 2 in the main paper. Next to the largest overlap of 587 proteins between resources mainly collecting dbTF proteins, there is a substantial overlap (311 proteins) between the two largest resources focussing coTFs (AnimalTFDB-coTF and TcoF-coTF), and also a noteworthy overlap of all three resources focussing on coTFs (91 proteins in all of GO-coTF, AnimalTFDB-coTF and TcoF-coTF).

Transcription factor info

Download

Gene Symbol	Gene Name	Synonyms	Human	Mouse	Rat
AHDC1	DJ159A19.3 RP1-159A19.1	Transcription factor Gibbin	27245	NA	NA

GO annotation and evidences

GO ID	GO:0140223	GO:0003700	GO:0003712	GO:0043565	GO:0006357	GO:0000981
**Annotation**	NA	QuickGO	NA	NA	NA	NA
**Evidence**	NA	Experimental evidence used in manual assertion	NA	NA	NA	NA

Other IDs

	Human	Mouse	Rat
**UniProtKB IDs**	Q5TGY3	NA	NA
**Ensembl IDs**	ENSG00000126705	NA	NA

Sources

AnimalTFDB_Human_dbTF	AnimalTFDB_Human_coTF	AnimalTFDB_Mouse_dbTF	AnimalTFDB_Mouse_coTF	AnimalTFDB_Rat_dbTF	AnimalTFDB_Rat_coTF
-	-	-	-	-	-
**DBD**	**JASPAR_Human**	**JASPAR_Mouse**	**JASPAR_Rat**	**Lambert & Jolma**	**Lovering**
-	-	-	-	AHDC1	-
**ORFeome**	**Ravasi**	**TcoF_Human_dbTF**	**TcoF_Human_coTF**	**TcoF_Mouse_dbTF**	**TcoF_Mouse_coTF**
-	-	-	-	-	-
**Saeed**	**TFCat**	**TFClass Human**	**TFClass Mouse**	**TFClass Rat**	**Vaquerizas**
-	-	-	-	-	✓(x)

Figure S3: Transcription factor info page for AHDC1: On this page, users can check if the protein of interest - in this case, AHDC1 - has human, mouse and rat orthologs. For this protein, there is no mouse or rat ortholog. Users can also check the GO annotation status of the protein (GO:0140223, ‘general transcription initiation factor activity’; GO:0003700, ‘DNA-binding transcription factor activity’; GO:0003712, ‘transcription coregulator activity’; GO:0043565, ‘sequence-specific DNA binding’; GO:0006357, ‘regulation of transcription by RNA polymerase II’ and GO:0000981, ‘DNA-binding transcription factor activity, RNA polymerase II-specific’). AHDC1 is annotated with GO term GO:0003700, indicating that it has transcription factor activity, as supported by experimental evidence. In addition to official symbols and Gene IDs, other identifiers are also available for AHDC1 in this page. The presence or absence of the protein of interest in the individual TFCheckpoint sources is also shown in this page; AHDC1, for example, is listed in the Lambert&Jolma and Vaquerizas collections of dbTFs; in the Vaquerizas collection, this protein is classified as ‘unlikely TF’, as indicated by “(x)”. When a link-out to the original source is available, the gene symbol is shown under the source name; for AHDC1, a link-out is available for Lambert&Jolma.

Transcription factor info

Download

Gene Symbol	Gene Name	Synonyms	Human	Mouse	Rat
GTF2E2	TFIIE-B FE TF2E2	Transcription initiation factor IIE subunit beta	2961	68153	306516

GO annotation and evidences

GO ID	GO:0140223	GO:0003700	GO:0003712	GO:0043565	GO:0006357	GO:0000981
**Annotation**	QuickGO	NA	NA	NA	NA	NA
**Evidence**	Experimental evidence used in manual assertion	NA	NA	NA	NA	NA

Other IDs

	Human	Mouse	Rat
**UniProtKB IDs**	P29084	Q9D902	D3ZCP9
**Ensembl IDs**	ENSG00000197265	ENSMUSG00000031585	ENSRNOG00000014422

Sources

AnimalTFDB_Human_dbTF	AnimalTFDB_Human_coTF	AnimalTFDB_Mouse_dbTF	AnimalTFDB_Mouse_coTF	AnimalTFDB_Rat_dbTF	AnimalTFDB_Rat_coTF
-	-	-	-	-	-
**DBD**	**JASPAR_Human**	**JASPAR_Mouse**	**JASPAR_Rat**	**Lambert & Jolma**	**Lovering**
-	-	-	-	-	-
**ORFeome**	**Ravasi**	**TcoF_Human_dbTF**	**TcoF_Human_coTF**	**TcoF_Mouse_dbTF**	**TcoF_Mouse_coTF**
✓	✓	-	-	-	-
**Saeed**	**TFCat**	**TFClass Human**	**TFClass Mouse**	**TFClass Rat**	**Vaquerizas**
-	✓	-	-	-	✓(x)

Figure S4: Transcription factor info page for GTF2E2. Users can observe that GTF2E2 has human, mouse and rat orthologs. Users can also check the GO annotation status of GTF2E2 regarding transcription regulation-related GO terms. According to GO, GTF2E2 has general transcription factor activity (GO:0140223) that is supported by experimental evidence. In addition to official symbols and Gene IDs, other identifiers are also available for all GTF2E2 orthologs (section 'Other IDs'). The presence or absence in the individual TFCheckpoint sources is also indicated: GTF2E2 is listed in the TFCat, Ravasi, ORFeome and Vaquerizas collections; in the Vaquerizas collection, this protein is classified as unlikely dbTF as indicated by "(x)".

Transcription factor info

Download

Gene Symbol	Gene Name	Synonyms	Human	Mouse	Rat
BDP1	TFIIIB150 TFC5 TFIIIB90 KIAA1689 HSA238520 KIAA1241	Transcription factor TFIIIB component B'' homolog	55814	544971	294687

GO annotation and evidences

GO ID	GO:0140223	GO:0003700	GO:0003712	GO:0043565	GO:0006357	GO:0000981
**Annotation**	NA	NA	NA	NA	NA	NA
**Evidence**	NA	NA	NA	NA	NA	NA

Other IDs

	Human	Mouse	Rat
**UniProtKB IDs**	A6H8Y1	Q571C7	B2RZ11
**Ensembl IDs**	ENSG00000145734	ENSMUSG00000049658	ENSRNOG00000017864

Sources

AnimalTFDB_Human_dbTF	AnimalTFDB_Human_coTF	AnimalTFDB_Mouse_dbTF	AnimalTFDB_Mouse_coTF	AnimalTFDB_Rat_dbTF	AnimalTFDB_Rat_coTF
-	BDP1	-	BDP1	-	BDP1
**DBD**	**JASPAR_Human**	**JASPAR_Mouse**	**JASPAR_Rat**	**Lambert & Jolma**	**Lovering**
✓	-	-	-	-	-
**ORFeome**	**Ravasi**	**TcoF_Human_dbTF**	**TcoF_Human_coTF**	**TcoF_Mouse_dbTF**	**TcoF_Mouse_coTF**
-	-	-	-	-	-
**Saeed**	**TFCat**	**TFClass Human**	**TFClass Mouse**	**TFClass Rat**	**Vaquerizas**
-	✓	-	-	-	✓(x)

Figure S5: Transcription factor info page for BDP1. The user can observe that BDP1 has human, mouse and rat orthologs. However, BDP1 is not associated with any GO annotation. In addition to official symbols and Gene IDs, other identifiers are also available for all BDP1 orthologs in this page. Furthermore, BDP1 is listed in the coTF section of AnimalTFDB (including all orthologs), and in the DBD, TFCat and Vaquerizas collection (classified as 'unlikely TF', marked with (x)) of TFs. For BDP1, link-outs are available for human, mouse and rat orthologs in AnimalTFDB.

SUPPLEMENTARY TABLES

Table S1
List of GO terms that were used to fetch proteins with transcription factor activity from the GO database, version 30.05.23.. In the “Purpose” column, “Source” means retrieval of a protein functional class; “Annotation” means additional functional information shown in TFC2.

**GO ID**	**Aspect**	**GO Term**	**Purpose**
GO:0043565	Molecular Function	sequence-specific DNA binding	Annotation
GO:0006357	Biological Process	regulation of transcription by RNA polymerase II	Annotation
GO:0003700	Molecular Function	DNA-binding transcription factor activity	Source
GO:0000981	Molecular Function	DNA-binding transcription factor activity, RNA polymerase II-specific	Annotation
GO:0003712	Molecular Function	transcription coregulator activity	Source
GO:0140223	Molecular Function	general transcription initiation factor activity	Source

Table S2
SPARQL Query used to retrieve human, mouse and rat orthologs from Orthodb (v10.1). Pasting this text in the query interface at https://sparql.orthodb.org/ will generate the table with mouse, human and rat orthologs.

```
prefix : <http://purl.orthodb.org/>
select *
where {
?og a :OrthoGroup.
?og :ogBuiltAt taxon:40674.
?gene_m a :Gene.
?gene_h a :Gene.
?gene_r a :Gene.
?gene_m up:organism/a taxon:10090.
?gene_h up:organism/a taxon:9606.
?gene_r up:organism/a taxon:10116.
?gene_m :memberOf ?og.
?gene_h :memberOf ?og.
?gene_r :memberOf ?og.
?gene_m :name ?gene_m_gid.
?gene_h :name ?gene_h_gid.
```

```
?gene_r :name ?gene_r_gid.
}
```

Table S3

Mouse proteins (and their rat orthologs, when applicable) which are not accompanied by human orthologs.

Gene Name	Entrez Taxa ID	Entrez Gene ID	UniProt SwissProt	Ensembl Gene ID
A430033K04Rik	10090|10116	243308|304336	E9Q8G5|A0A0G2K1C7	ENSMUSG00000056014|ENSRNOG00000028651
Aicda	10090	11628	Q9WVE0	ENSMUSG00000040627
Arap1	10090	69710	Q4LDD4	ENSMUSG00000032812
Arglu1	10090|10116	234023|290912	Q3UL36|Q5BJT0	ENSMUSG00000040459|ENSRNOG00000024142
Arhgap22	10090	239027	Q8BL80	ENSMUSG00000063506
Asf1a	10090|10116	66403|294408	Q9CQE6|D3ZFM1	ENSMUSG00000019857|ENSRNOG00000000415
Atp5f1c	10116	116550	P35435	NA
Basp1	10090|10116	70350|64160	Q91XV3|Q05175	ENSMUSG00000045763|ENSRNOG00000046313
Borcs8	10090	72368	Q9D6Y4	ENSMUSG00000002345
Btn1a1	10090|10116	12231|306956	Q62556|F1LRV5	ENSMUSG00000000706|ENSRNOG00000017514
Cdc42bpg	10090	240505	Q80UW5	ENSMUSG00000024769
Chmp1a	10090|10116	234852|365024	Q921W0|D4AE79	ENSMUSG00000000743|ENSRNOG00000016001
Cmtm2a	10090|10116	73381|307616	Q9DAR1|Q6AYN6	ENSMUSG00000074127|ENSRNOG00000025420
Cphx3	10090|10116	105594|502042	Q8BX39|A0A0G2JZG9	ENSMUSG00000094817|ENSRNOG00000025433
Csprs	10090	114564	Q99388	ENSMUSG00000062783
Cys1	10090|10116	12879|690489	Q8R4T1|D3ZCZ3	ENSMUSG00000062563|ENSRNOG00000058891
Dpy30	10090|10116	66310|286897	Q99LT0|Q8K3E7	ENSMUSG00000024067|ENSRNOG00000027126
Ecsit	10090|10116	26940|300447	Q9QZH6|Q5XIC2	ENSMUSG00000066839|ENSRNOG00000014128
Eid3	10090	66341	Q3V124	ENSMUSG00000109864
Eif4a3	10090|10116	192170|688288	Q91VC3|Q3B8Q2	ENSMUSG00000025580|ENSRNOG00000045791
Elob	10090|10116	67673|81807	P62869|P62870	ENSMUSG00000055839|ENSRNOG00000004814
Elp4	10090|10116	77766|687694	Q9ER73|B0BN35	ENSMUSG00000027167|
Fgf11	10090	14166	P70378	ENSMUSG00000042826
Fgf2	10090|10116	14173|54250	P15655|P13109	ENSMUSG00000037225|ENSRNOG00000017392
Gnas	10090|10116	14683|24896	P63094|Q9Z213	ENSMUSG00000027523|ENSRNOG00000025889
Gtf2a1l	10090|10116	71828|316711	Q8R4I4|Q641W8	ENSMUSG00000024154|ENSRNOG00000016703
Hdgfl3	10090|10116	29877|252941	Q9JMG7|Q923W4	ENSMUSG00000025104|ENSRNOG00000019740
Hsf3	10090	245525	D0VYS2	ENSMUSG00000045802
Ikbkg	10090|10116	16151|309295	O88522|Q6TMG5	ENSMUSG00000004221|ENSRNOG00000060936
Med29	10090|10116	67224|292751	Q9DB91|D4A108	ENSMUSG00000003444|ENSRNOG00000019702
Msx3	10090|10116	17703|114504	P70354|G3V8C7	ENSMUSG00000025469|ENSRNOG00000046776
Mycb	10090|10116	107771|311807	Q6P8Z1|P15063	ENSMUSG00000049086|ENSRNOG00000042556
Ndufa13	10090|10116	67184|100911483	Q9ERS2|D3ZE15	ENSMUSG00000036199|ENSRNOG00000020602
Npm2	10090|10116	328440|290359	Q80W85|Q7M6Z1	ENSMUSG00000047911|ENSRNOG00000025638
Nsmce3	10090|10116	66647|309259	Q9CPR8|Q4KM72	ENSMUSG00000070520|
Nup62	10090|10116	18226|65274	Q63850|P17955	ENSMUSG00000109511|ENSRNOG00000048733
P2rx2	10090	231602	Q8K3P1	ENSMUSG00000029503
Pak6	10090|10116	214230|296078	Q3ULB5|D3ZQ51	ENSMUSG00000074923|ENSRNOG00000007925
Ppp1r15a	10090|10116	17872|103691034	P17564|Q6IN02	ENSMUSG00000040435|ENSRNOG00000014791
Pus1	10090|10116	56361|304567	Q9WU56|Q4KM92	ENSMUSG00000029507|ENSRNOG00000037500
Rad54l2	10090|10116	81000|363135	Q99NG0|D3ZV87	ENSMUSG00000040661|ENSRNOG00000013570
Retreg1	10116	103689968	Q5FVM3	ENSRNOG00000010589
Rhox13	10090|10116	73614|691244	F6YCR7|F1M524	ENSMUSG00000050197|ENSRNOG00000028050
Rhox5	10090|10116	18617|24631	P52651|Q63630	ENSMUSG00000095180|ENSRNOG00000046548
Ripply1	10090|10116	622473|680702	Q2WG77|A0A0G2K721	ENSMUSG00000072945|ENSRNOG00000061101
Rnf141	10090|10116	67150|308900	Q99MB7|Q6IV57	ENSMUSG00000030788|ENSRNOG00000017900
Rpf2	10090	67239	Q9JJ80	ENSMUSG00000038510

Rpl11	10090|10116	67025|362631	Q9CXW4|P62914	ENSMUSG00000059291|ENSRNOG00000026260
Setdb2	10090|10116	239122|100361710	Q8C267|M0R6H5	ENSMUSG00000071350|ENSRNOG00000021680
Sohlh2	10090|10116	74434|619575	Q9D489|Q3MHT3	ENSMUSG00000027794|ENSRNOG00000038091
Spop	10090|10116	20747|287643	Q6ZWS8|B2RYD9	ENSMUSG00000057522|ENSRNOG00000004686
Srst	10090	21331	Q06666	ENSMUSG00000058159
TUT7	10090	214290	Q5BLK4	NA
Tnp1	10090|10116	21958|24839	P10856|P02317	ENSMUSG00000026182|ENSRNOG00000017611
Traf7	10090|10116	224619|360491	Q922B6|B1WBW7	ENSMUSG00000052752|ENSRNOG00000003131
Treml1	10090	71326	Q8K558	ENSMUSG00000023993
Trib2	10090	217410	Q8K4K3	ENSMUSG00000020601
Tsc22d4	10090|10116	78829|100362783	Q9EQN3|Q3B8N7	ENSMUSG00000029723|ENSRNOG00000024616
Ube2i	10090|10116	22196|25573	P63280|P63281	ENSMUSG00000015120|ENSRNOG00000017907
Vhl	10090|10116	22346|24874	P40338|Q64259	ENSMUSG00000033933|ENSRNOG00000010258
Wtip	10090	101543	Q7TQJ8	ENSMUSG00000036459
Zfp11	10090|10116	22648|100363065	P10751|A0A0G2K5Y5	ENSMUSG00000051034|ENSRNOG00000051442
Zfp120	10090|10116	104348|102554315	Q8BZW4|F1LWY5	ENSMUSG00000068134|ENSRNOG00000006112
Zfp54	10090|10116	22712|308232	E9PW05|B5DEZ5	ENSMUSG00000023882|ENSRNOG00000051578
Zfp563	10090|10116	240068|314584	Q62516|F1M4J4	ENSMUSG00000067424|ENSRNOG00000030273
Zfp58	10090	238693	P16372	ENSMUSG00000071291
Zfp809	10090	235047	G3X9G7	ENSMUSG00000057982
Zfp84	10090|10116	74352|308482	Q9D654|M0R490	ENSMUSG00000046185|ENSRNOG00000029043
Zfp955a	10090|10116	100043468|314600	Q80XR7|A0A0G2K0K0	ENSMUSG00000096910|ENSRNOG00000048577
Zfy1	10090|10116	22767|103694540	P10925|A0A0G2K3V6	ENSMUSG00000053211|ENSRNOG00000053042
Zfy2	10090|10116	22768|103694540	P20662|A0A0G2K3V6	ENSMUSG00000000103|ENSRNOG00000053042
Zim1	10090|10116	22776|308322	Q8C393|A0A0G2KA26	ENSMUSG00000002266|ENSRNOG00000015071
Znf239	10090|10116	22685|100909657	P24399|M0RBC3	ENSMUSG00000042097|ENSRNOG00000014552
Znf271	10090|10116	22694|307547	P15620|Q5FVP4	ENSMUSG00000063281|ENSRNOG00000049137
Znf354c	10090|10116	30944|78972	Q571J5|Q9EPU7	ENSMUSG00000044807|ENSRNOG00000029205
Znf431	10090	69504	E9QAG8	ENSMUSG00000066613
Znf568	10090	243905	E9PYI1	ENSMUSG00000074221
Znf667	10090|10116	384763|308326	Q2TL60|Q5MYW4	ENSMUSG00000054893|ENSRNOG00000033906
Znf728	10090|10116	238690|499563	Q6P5C7|Q5U307	ENSMUSG00000055480|ENSRNOG00000017986
Znf768	10090|10116	233890|102553763	Q8R0T2|D3ZGK0	ENSMUSG00000047371|ENSRNOG00000029490

Table S4
Proteins with recorded evidence according to Gene Ontology (as of 30.05.2023).

GO-dbTF (as of 30.05.2023)				
**Protein**	**Experimental evidence**	**Phylogenetic evidence**	**Manual computational evidence**	**Automatic computational evidence**
ADNP	1	0	0	0
AEBP1	1	0	0	0
**AFF1**	**1**	0	0	0
AFF3	1	0	0	0
AHDC1	1	0	0	0
AHR	1	0	0	0

ALX1	1	0	0	0
ALX4	1	0	0	0
AR	1	0	0	0
ARNT	1	0	0	0
ARNT2	1	0	0	0
ARNTL	1	0	0	0
ARNTL2	1	0	0	0
ARX	1	0	0	0
ASCL1	1	0	0	0
ASCL2	1	0	0	0
ASCL3	1	0	0	0
ATF1	1	0	0	0
ATF2	1	0	0	0
ATF3	1	0	0	0
ATF4	1	0	0	0
ATF5	1	0	0	0
ATF6	1	0	0	0
ATF6B	1	0	0	0
ATF7	1	0	0	0
ATMIN	1	0	0	0
ATOH1	1	0	0	0
ATOH8	1	0	0	0
BACH1	1	0	0	0
BARHL1	1	0	0	0
BARHL2	1	0	0	0
BARX2	1	0	0	0
BATF	1	0	0	0
BATF3	1	0	0	0
BCL11A	1	0	0	0
BCL11B	1	0	0	0
BCL6	1	0	0	0
BCL6B	1	0	0	0
BHLHA15	1	0	0	0
BHLHE22	1	0	0	0

BHLHE40	1	0	0	0
BHLHE41	1	0	0	0
BNC1	1	0	0	0
BSX	1	0	0	0
CARF	1	0	0	0
CASZ1	1	0	0	0
CC2D1A	1	0	0	0
CC2D1B	1	0	0	0
CDC5L	1	0	0	0
CDX1	1	0	0	0
CDX2	1	0	0	0
CDX4	1	0	0	0
CEBPA	1	0	0	0
CEBPB	1	0	0	0
CEBPD	1	0	0	0
CEBPE	1	0	0	0
CEBPG	1	0	0	0
CIAO1	1	0	0	0
CLOCK	1	0	0	0
CREB1	1	0	0	0
CREB3	1	0	0	0
CREB3L1	1	0	0	0
CREB3L2	1	0	0	0
CREB3L3	1	0	0	0
CREB3L4	1	0	0	0
CREB5	1	0	0	0
CREBRF	1	0	0	0
CREM	1	0	0	0
CRX	1	0	0	0
CSRNP1	1	0	0	0
CSRNP2	1	0	0	0
CSRNP3	1	0	0	0
CTCF	1	0	0	0
CTCFL	1	0	0	0

CUX2	1	0	0	0
DACH1	1	0	0	0
DACH2	1	0	0	0
DBP	1	0	0	0
DDIT3	1	0	0	0
DDN	1	0	0	0
DEAF1	1	0	0	0
DLX1	1	0	0	0
DLX2	1	0	0	0
DLX3	1	0	0	0
DLX4	1	0	0	0
DLX5	1	0	0	0
DMBX1	1	0	0	0
DMRT1	1	0	0	0
DMRT2	1	0	0	0
DMTF1	1	0	0	0
DUX1	1	0	0	0
DUX4	1	0	0	0
E2F1	1	0	0	0
E2F2	1	0	0	0
E2F3	1	0	0	0
E2F4	1	0	0	0
E2F5	1	0	0	0
E2F6	1	0	0	0
E2F7	1	0	0	0
E2F8	1	0	0	0
E4F1	1	0	0	0
EBF1	1	0	0	0
EBF2	1	0	0	0
EBF3	1	0	0	0
EBF4	1	0	0	0
EGR1	1	0	0	0
EGR2	1	0	0	0
EGR3	1	0	0	0

EGR4	1	0	0	0
EHF	1	0	0	0
ELF1	1	0	0	0
ELF2	1	0	0	0
ELF3	1	0	0	0
ELF4	1	0	0	0
ELF5	1	0	0	0
ELK1	1	0	0	0
ELK3	1	0	0	0
ELK4	1	0	0	0
EN1	1	0	0	0
ENO1	1	0	0	0
EPAS1	1	0	0	0
ERF	1	0	0	0
ERG	1	0	0	0
ESR1	1	0	0	0
ESR2	1	0	0	0
ESRRA	1	0	0	0
ESRRB	1	0	0	0
ESRRG	1	0	0	0
ESX1	1	0	0	0
ETS1	1	0	0	0
ETS2	1	0	0	0
ETV1	1	0	0	0
ETV2	1	0	0	0
ETV3	1	0	0	0
ETV4	1	0	0	0
ETV5	1	0	0	0
ETV6	1	0	0	0
ETV7	1	0	0	0
Ecsit	1	0	0	0
FERD3L	1	0	0	0
FEV	1	0	0	0
FEZF1	1	0	0	0

FEZF2	1	0	0	0
FIGLA	1	0	0	0
FLI1	1	0	0	0
FOS	1	0	0	0
FOSB	1	0	0	0
FOSL1	1	0	0	0
FOSL2	1	0	0	0
FOXA1	1	0	0	0
FOXA2	1	0	0	0
FOXA3	1	0	0	0
FOXC1	1	0	0	0
FOXC2	1	0	0	0
FOXD1	1	0	0	0
FOXD2	1	0	0	0
FOXD3	1	0	0	0
FOXD4	1	0	0	0
FOXD4L1	1	0	0	0
FOXD4L3	1	0	0	0
FOXE1	1	0	0	0
FOXE3	1	0	0	0
FOXF1	1	0	0	0
FOXF2	1	0	0	0
FOXH1	1	0	0	0
FOXI1	1	0	0	0
FOXJ1	1	0	0	0
FOXJ2	1	0	0	0
FOXJ3	1	0	0	0
FOXK1	1	0	0	0
FOXK2	1	0	0	0
FOXL1	1	0	0	0
FOXL2	1	0	0	0
FOXM1	1	0	0	0
FOXN1	1	0	0	0
FOXN4	1	0	0	0

FOXO1	1	0	0	0
FOXO3	1	0	0	0
FOXO4	1	0	0	0
FOXO6	1	0	0	0
FOXP1	1	0	0	0
FOXP2	1	0	0	0
FOXP3	1	0	0	0
FOXP4	1	0	0	0
FOXQ1	1	0	0	0
FOXR1	1	0	0	0
FOXS1	1	0	0	0
GABPA	1	0	0	0
GATA1	1	0	0	0
GATA2	1	0	0	0
GATA3	1	0	0	0
GATA4	1	0	0	0
GATA5	1	0	0	0
GATA6	1	0	0	0
GBX2	1	0	0	0
GCM1	1	0	0	0
GCM2	1	0	0	0
GFI1	1	0	0	0
GLI1	1	0	0	0
GLI2	1	0	0	0
GLI3	1	0	0	0
GLIS1	1	0	0	0
GLIS2	1	0	0	0
GLIS3	1	0	0	0
GMEB1	1	0	0	0
GMEB2	1	0	0	0
GPBP1	1	0	0	0
GRHL1	1	0	0	0
GRHL2	1	0	0	0
GRHL3	1	0	0	0

GSC	1	0	0	0
GSC2	1	0	0	0
GSX1	1	0	0	0
GSX2	1	0	0	0
GTF2I	1	0	0	0
GTF2IRD1	1	0	0	0
GTF2IRD2	1	0	0	0
GTF2IRD2B	1	0	0	0
GZF1	1	0	0	0
HAND1	1	0	0	0
HAND2	1	0	0	0
HBP1	1	0	0	0
HDAC5	1	0	0	0
HDGF	1	0	0	0
HELT	1	0	0	0
HES1	1	0	0	0
HES2	1	0	0	0
HES3	1	0	0	0
HES5	1	0	0	0
HES6	1	0	0	0
HESX1	1	0	0	0
HEY1	1	0	0	0
HEY2	1	0	0	0
HEYL	1	0	0	0
HHEX	1	0	0	0
HIC1	1	0	0	0
HIF1A	1	0	0	0
HIF3A	1	0	0	0
HINFP	1	0	0	0
HIVEP2	1	0	0	0
HLF	1	0	0	0
HMX1	1	0	0	0
HNF1A	1	0	0	0
HNF1B	1	0	0	0

HNF4A	1	0	0	0
HNF4G	1	0	0	0
HOXA1	1	0	0	0
HOXA10	1	0	0	0
HOXA13	1	0	0	0
HOXA2	1	0	0	0
HOXA3	1	0	0	0
HOXA5	1	0	0	0
HOXA7	1	0	0	0
HOXA9	1	0	0	0
HOXB1	1	0	0	0
HOXB13	1	0	0	0
HOXB2	1	0	0	0
HOXB3	1	0	0	0
HOXB5	1	0	0	0
HOXB7	1	0	0	0
HOXB8	1	0	0	0
HOXC10	1	0	0	0
HOXC11	1	0	0	0
HOXC13	1	0	0	0
HOXC4	1	0	0	0
HOXD10	1	0	0	0
HOXD13	1	0	0	0
HOXD3	1	0	0	0
HOXD4	1	0	0	0
HOXD8	1	0	0	0
HOXD9	1	0	0	0
HSF1	1	0	0	0
HSF2	1	0	0	0
HSF4	1	0	0	0
IKZF1	1	0	0	0
IKZF3	1	0	0	0
IKZF5	1	0	0	0
ILF3	1	0	0	0

INSM1	1	0	0	0
IRF1	1	0	0	0
IRF2	1	0	0	0
IRF3	1	0	0	0
IRF4	1	0	0	0
IRF5	1	0	0	0
IRF6	1	0	0	0
IRF7	1	0	0	0
IRF8	1	0	0	0
IRX1	1	0	0	0
IRX2	1	0	0	0
IRX3	1	0	0	0
IRX6	1	0	0	0
ISL1	1	0	0	0
ISX	1	0	0	0
JDP2	1	0	0	0
JUN	1	0	0	0
JUNB	1	0	0	0
JUND	1	0	0	0
KCNIP3	1	0	0	0
KLF1	1	0	0	0
KLF10	1	0	0	0
KLF11	1	0	0	0
KLF12	1	0	0	0
KLF13	1	0	0	0
KLF15	1	0	0	0
KLF16	1	0	0	0
KLF17	1	0	0	0
KLF2	1	0	0	0
KLF4	1	0	0	0
KLF5	1	0	0	0
KLF6	1	0	0	0
KLF7	1	0	0	0
KLF8	1	0	0	0

KLF9	1	0	0	0
KMT2D	1	0	0	0
LBX1	1	0	0	0
LEF1	1	0	0	0
LHX1	1	0	0	0
LHX2	1	0	0	0
LHX3	1	0	0	0
LHX4	1	0	0	0
LHX5	1	0	0	0
LITAF	1	0	0	0
LMX1A	1	0	0	0
LMX1B	1	0	0	0
LRRFIP1	1	0	0	0
MAF	1	0	0	0
MAFA	1	0	0	0
MAFB	1	0	0	0
MAFF	1	0	0	0
MAFG	1	0	0	0
MAFK	1	0	0	0
MAX	1	0	0	0
MAZ	1	0	0	0
MECOM	1	0	0	0
MECP2	1	0	0	0
MEF2A	1	0	0	0
MEF2B	1	0	0	0
MEF2C	1	0	0	0
MEF2D	1	0	0	0
MEIS1	1	0	0	0
MEIS2	1	0	0	0
MEIS3	1	0	0	0
MEN1	1	0	0	0
MEOX1	1	0	0	0
MEOX2	1	0	0	0
MESP1	1	0	0	0

MESP2	1	0	0	0
MGA	1	0	0	0
MITF	1	0	0	0
MIXL1	1	0	0	0
MKX	1	0	0	0
MLX	1	0	0	0
MLXIP	1	0	0	0
MLXIPL	1	0	0	0
MNT	1	0	0	0
MRTFA	1	0	0	0
MRTFB	1	0	0	0
MSC	1	0	0	0
MSGN1	1	0	0	0
MSX1	1	0	0	0
MSX2	1	0	0	0
MTF1	1	0	0	0
MTF2	1	0	0	0
MXD1	1	0	0	0
MXD3	1	0	0	0
MXD4	1	0	0	0
MXI1	1	0	0	0
MYB	1	0	0	0
MYBL1	1	0	0	0
MYBL2	1	0	0	0
MYC	1	0	0	0
MYCN	1	0	0	0
MYEF2	1	0	0	0
MYF5	1	0	0	0
MYF6	1	0	0	0
MYOD1	1	0	0	0
MYOG	1	0	0	0
MYPOP	1	0	0	0
MYRF	1	0	0	0
MYT1	1	0	0	0

MYT1L	1	0	0	0
MZF1	1	0	0	0
NACC2	1	0	0	0
NANOG	1	0	0	0
NANOGP8	1	0	0	0
NCOA3	1	0	0	0
NDN	1	0	0	0
NEUROD1	1	0	0	0
NEUROD2	1	0	0	0
NEUROD6	1	0	0	0
NEUROG3	1	0	0	0
NFAT5	1	0	0	0
NFATC1	1	0	0	0
NFATC2	1	0	0	0
NFATC3	1	0	0	0
NFATC4	1	0	0	0
NFE2L1	1	0	0	0
NFE2L2	1	0	0	0
NFE2L3	1	0	0	0
NFIA	1	0	0	0
NFIB	1	0	0	0
NFIC	1	0	0	0
NFIL3	1	0	0	0
NFIX	1	0	0	0
NFKB1	1	0	0	0
NFKB2	1	0	0	0
NFX1	1	0	0	0
NFYA	1	0	0	0
NFYB	1	0	0	0
NFYC	1	0	0	0
NHLH1	1	0	0	0
NHLH2	1	0	0	0
NKRF	1	0	0	0
NKX2-1	1	0	0	0

NKX2-2	1	0	0	0
NKX2-3	1	0	0	0
NKX2-4	1	0	0	0
NKX2-5	1	0	0	0
NKX2-6	1	0	0	0
NKX2-8	1	0	0	0
NKX3-1	1	0	0	0
NKX3-2	1	0	0	0
NKX6-1	1	0	0	0
NKX6-2	1	0	0	0
NKX6-3	1	0	0	0
NOBOX	1	0	0	0
NPAS2	1	0	0	0
NPAS4	1	0	0	0
NR1D1	1	0	0	0
NR1D2	1	0	0	0
NR1H2	1	0	0	0
NR1H3	1	0	0	0
NR1H4	1	0	0	0
NR1I2	1	0	0	0
NR1I3	1	0	0	0
NR2C1	1	0	0	0
NR2C2	1	0	0	0
NR2E1	1	0	0	0
NR2E3	1	0	0	0
NR2F1	1	0	0	0
NR2F2	1	0	0	0
NR2F6	1	0	0	0
NR3C1	1	0	0	0
NR3C2	1	0	0	0
NR4A1	1	0	0	0
NR4A2	1	0	0	0
NR4A3	1	0	0	0
NR5A1	1	0	0	0

NR5A2	1	0	0	0
NR6A1	1	0	0	0
NRF1	1	0	0	0
NRL	1	0	0	0
OLIG2	1	0	0	0
ONECUT1	1	0	0	0
ONECUT2	1	0	0	0
ONECUT3	1	0	0	0
OSR2	1	0	0	0
OTP	1	0	0	0
OTX1	1	0	0	0
OTX2	1	0	0	0
OVOL1	1	0	0	0
OVOL2	1	0	0	0
PAX1	1	0	0	0
PAX2	1	0	0	0
PAX3	1	0	0	0
PAX4	1	0	0	0
PAX5	1	0	0	0
PAX6	1	0	0	0
PAX7	1	0	0	0
PAX8	1	0	0	0
PBX1	1	0	0	0
PBX2	1	0	0	0
PBX3	1	0	0	0
PCBP1	1	0	0	0
PCBP3	1	0	0	0
PDX1	1	0	0	0
PGR	1	0	0	0
PHOX2A	1	0	0	0
PHOX2B	1	0	0	0
PITX1	1	0	0	0
PITX2	1	0	0	0
PITX3	1	0	0	0

PKNOX1	1	0	0	0
PLAG1	1	0	0	0
PLAGL1	1	0	0	0
PLAGL2	1	0	0	0
PLSCR1	1	0	0	0
POU1F1	1	0	0	0
POU2F1	1	0	0	0
POU2F2	1	0	0	0
POU2F3	1	0	0	0
POU3F1	1	0	0	0
POU3F2	1	0	0	0
POU3F3	1	0	0	0
POU3F4	1	0	0	0
POU4F1	1	0	0	0
POU4F2	1	0	0	0
POU4F3	1	0	0	0
POU5F1	1	0	0	0
POU5F1B	1	0	0	0
POU6F1	1	0	0	0
PPARA	1	0	0	0
PPARD	1	0	0	0
PPARG	1	0	0	0
PRDM1	1	0	0	0
PRDM14	1	0	0	0
PRDM16	1	0	0	0
PRDM2	1	0	0	0
PRDM4	1	0	0	0
PRDM5	1	0	0	0
PROP1	1	0	0	0
PROX1	1	0	0	0
PRRX1	1	0	0	0
PRRX2	1	0	0	0
PTF1A	1	0	0	0
PURA	1	0	0	0

PURB	1	0	0	0
RARA	1	0	0	0
RARB	1	0	0	0
RARG	1	0	0	0
RAX	1	0	0	0
RAX2	1	0	0	0
RBPJ	1	0	0	0
RBPJL	1	0	0	0
REL	1	0	0	0
RELA	1	0	0	0
REST	1	0	0	0
RFX2	1	0	0	0
RFX3	1	0	0	0
RFX4	1	0	0	0
RFX5	1	0	0	0
RFX6	1	0	0	0
RFXANK	1	0	0	0
RFXAP	1	0	0	0
RORA	1	0	0	0
RORB	1	0	0	0
RORC	1	0	0	0
RREB1	1	0	0	0
RUNX1	1	0	0	0
RUNX2	1	0	0	0
RUNX3	1	0	0	0
RXRA	1	0	0	0
RXRB	1	0	0	0
RXRG	1	0	0	0
Rhox5	1	0	0	0
SALL1	1	0	0	0
SALL2	1	0	0	0
SATB1	1	0	0	0
SATB2	1	0	0	0
SCRT1	1	0	0	0

SCX	1	0	0	0
SHOX	1	0	0	0
SIX1	1	0	0	0
SIX2	1	0	0	0
SIX3	1	0	0	0
SIX4	1	0	0	0
SIX5	1	0	0	0
SKI	1	0	0	0
SKIL	1	0	0	0
SLC2A4RG	1	0	0	0
SMAD1	1	0	0	0
SMAD2	1	0	0	0
SMAD3	1	0	0	0
SMAD4	1	0	0	0
SMAD5	1	0	0	0
SNAI1	1	0	0	0
SNAI2	1	0	0	0
SNAI3	1	0	0	0
SOHLH1	1	0	0	0
SOX1	1	0	0	0
SOX10	1	0	0	0
SOX11	1	0	0	0
SOX12	1	0	0	0
SOX13	1	0	0	0
SOX14	1	0	0	0
SOX15	1	0	0	0
SOX17	1	0	0	0
SOX18	1	0	0	0
SOX2	1	0	0	0
SOX21	1	0	0	0
SOX30	1	0	0	0
SOX4	1	0	0	0
SOX5	1	0	0	0
SOX6	1	0	0	0

SOX7	1	0	0	0
SOX8	1	0	0	0
SOX9	1	0	0	0
SP1	1	0	0	0
SP100	1	0	0	0
SP2	1	0	0	0
SP3	1	0	0	0
SP5	1	0	0	0
SP7	1	0	0	0
SPEN	1	0	0	0
SPI1	1	0	0	0
SPIB	1	0	0	0
SPIC	1	0	0	0
SPZ1	1	0	0	0
SREBF1	1	0	0	0
SREBF2	1	0	0	0
SRF	1	0	0	0
SRY	1	0	0	0
STAT1	1	0	0	0
STAT3	1	0	0	0
STAT4	1	0	0	0
STAT5A	1	0	0	0
STAT5B	1	0	0	0
STAT6	1	0	0	0
Sohlh2	1	0	0	0
TAL1	1	0	0	0
TBR1	1	0	0	0
TBX1	1	0	0	0
TBX15	1	0	0	0
TBX18	1	0	0	0
TBX19	1	0	0	0
TBX2	1	0	0	0
TBX20	1	0	0	0
TBX21	1	0	0	0

TBX3	1	0	0	0
TBX5	1	0	0	0
TBXT	1	0	0	0
TCF12	1	0	0	0
TCF15	1	0	0	0
TCF20	1	0	0	0
TCF21	1	0	0	0
TCF3	1	0	0	0
TCF4	1	0	0	0
TCF7	1	0	0	0
TCF7L1	1	0	0	0
TCF7L2	1	0	0	0
TCFL5	1	0	0	0
TEAD1	1	0	0	0
TEAD2	1	0	0	0
TEAD3	1	0	0	0
TEAD4	1	0	0	0
TEF	1	0	0	0
TFAP2A	1	0	0	0
TFAP2B	1	0	0	0
TFAP2C	1	0	0	0
TFAP2D	1	0	0	0
TFAP2E	1	0	0	0
TFAP4	1	0	0	0
TFCP2	1	0	0	0
TFDP1	1	0	0	0
TFDP2	1	0	0	0
TFDP3	1	0	0	0
TFE3	1	0	0	0
TFEB	1	0	0	0
TFEC	1	0	0	0
TGIF1	1	0	0	0
THAP1	1	0	0	0
THAP11	1	0	0	0

THRA	1	0	0	0
THRB	1	0	0	0
TLX2	1	0	0	0
TP53	1	0	0	0
TP63	1	0	0	0
TP73	1	0	0	0
TRPS1	1	0	0	0
TSC22D1	1	0	0	0
TWIST1	1	0	0	0
TWIST2	1	0	0	0
UBP1	1	0	0	0
USF1	1	0	0	0
USF2	1	0	0	0
USF3	1	0	0	0
VAX1	1	0	0	0
VAX2	1	0	0	0
VDR	1	0	0	0
VENTX	1	0	0	0
VEZF1	1	0	0	0
VSX2	1	0	0	0
WIZ	1	0	0	0
WT1	1	0	0	0
XBP1	1	0	0	0
YLPM1	1	0	0	0
YY1	1	0	0	0
YY2	1	0	0	0
ZBED1	1	0	0	0
ZBED2	1	0	0	0
ZBED4	1	0	0	0
ZBED6	1	0	0	0
ZBTB10	1	0	0	0
ZBTB14	1	0	0	0
ZBTB16	1	0	0	0
ZBTB17	1	0	0	0

ZBTB18	1	0	0	0
ZBTB2	1	0	0	0
ZBTB20	1	0	0	0
ZBTB21	1	0	0	0
ZBTB32	1	0	0	0
ZBTB38	1	0	0	0
ZBTB4	1	0	0	0
ZBTB46	1	0	0	0
ZBTB5	1	0	0	0
ZBTB7A	1	0	0	0
ZBTB7B	1	0	0	0
ZBTB8A	1	0	0	0
ZC3H8	1	0	0	0
ZEB1	1	0	0	0
ZEB2	1	0	0	0
ZFAT	1	0	0	0
ZFHX3	1	0	0	0
ZFP42	1	0	0	0
ZFP90	1	0	0	0
ZFX	1	0	0	0
ZGLP1	1	0	0	0
ZGPAT	1	0	0	0
ZHX1	1	0	0	0
ZHX3	1	0	0	0
ZIC1	1	0	0	0
ZIC2	1	0	0	0
ZIC3	1	0	0	0
ZIC5	1	0	0	0
ZKSCAN3	1	0	0	0
ZNF131	1	0	0	0
ZNF140	1	0	0	0
ZNF143	1	0	0	0
ZNF148	1	0	0	0
ZNF174	1	0	0	0

ZNF175	1	0	0	0
ZNF202	1	0	0	0
ZNF205	1	0	0	0
ZNF217	1	0	0	0
ZNF219	1	0	0	0
ZNF224	1	0	0	0
ZNF239	1	0	0	0
ZNF24	1	0	0	0
ZNF263	1	0	0	0
ZNF281	1	0	0	0
ZNF300	1	0	0	0
ZNF322	1	0	0	0
ZNF341	1	0	0	0
ZNF35	1	0	0	0
ZNF350	1	0	0	0
ZNF354A	1	0	0	0
ZNF354B	1	0	0	0
ZNF367	1	0	0	0
ZNF382	1	0	0	0
ZNF384	1	0	0	0
ZNF395	1	0	0	0
ZNF438	1	0	0	0
ZNF512B	1	0	0	0
ZNF536	1	0	0	0
ZNF579	1	0	0	0
ZNF589	1	0	0	0
ZNF618	1	0	0	0
ZNF628	1	0	0	0
ZNF639	1	0	0	0
ZNF644	1	0	0	0
ZNF668	1	0	0	0
ZNF692	1	0	0	0
ZNF746	1	0	0	0
ZNF750	1	0	0	0

ZNF76	1	0	0	0
ZNF85	1	0	0	0
ZNF91	1	0	0	0
ZNF93	1	0	0	0
ZSCAN10	1	0	0	0
ZSCAN21	1	0	0	0
AHRR	0	1	0	0
ALX3	0	1	0	0
ANHX	0	1	0	0
ARGFX	0	1	0	0
ASCL4	0	1	0	0
ASCL5	0	1	0	0
ATOH7	0	1	0	0
BACH2	0	1	0	0
BARX1	0	1	0	0
BATF2	0	1	0	0
BBX	0	1	0	0
BHLHA9	0	1	0	0
BHLHE23	0	1	0	0
CIC	0	1	0	0
CPHXL	0	1	0	0
CUX1	0	1	0	0
Csprs	0	1	0	0
DLX6	0	1	0	0
DMRT3	0	1	0	0
DMRTA1	0	1	0	0
DMRTA2	0	1	0	0
DMRTB1	0	1	0	0
DMRTC1B	0	1	0	0
DMRTC2	0	1	0	0
DPRX	0	1	0	0
DRGX	0	1	0	0
DUX3	0	1	0	0
DUX4L2	0	1	0	0

DUX4L3	0	1	0	0
DUX4L4	0	1	0	0
DUX4L5	0	1	0	0
DUX4L6	0	1	0	0
DUX4L7	0	1	0	0
DUX4L9	0	1	0	0
DUX5	0	1	0	0
DUXA	0	1	0	0
DUXB	0	1	0	0
EMX1	0	1	0	0
EMX2	0	1	0	0
EN2	0	1	0	0
EOMES	0	1	0	0
ERFL	0	1	0	0
ETV3L	0	1	0	0
EVX1	0	1	0	0
EVX2	0	1	0	0
FOXB1	0	1	0	0
FOXB2	0	1	0	0
FOXD4L4	0	1	0	0
FOXD4L5	0	1	0	0
FOXD4L6	0	1	0	0
FOXI2	0	1	0	0
FOXI3	0	1	0	0
FOXL3	0	1	0	0
FOXN2	0	1	0	0
FOXN3	0	1	0	0
GBX1	0	1	0	0
GFI1B	0	1	0	0
GLI4	0	1	0	0
HDX	0	1	0	0
HES4	0	1	0	0
HES7	0	1	0	0
HIC2	0	1	0	0

HIVEP1	0	1	0	0
HIVEP3	0	1	0	0
HMX2	0	1	0	0
HMX3	0	1	0	0
HOMEZ	0	1	0	0
HOXA11	0	1	0	0
HOXA4	0	1	0	0
HOXA6	0	1	0	0
HOXB4	0	1	0	0
HOXB6	0	1	0	0
HOXB9	0	1	0	0
HOXC5	0	1	0	0
HOXC6	0	1	0	0
HOXC8	0	1	0	0
HOXC9	0	1	0	0
HOXD1	0	1	0	0
HOXD11	0	1	0	0
HSF5	0	1	0	0
HSFX1	0	1	0	0
HSFX3	0	1	0	0
HSFX4	0	1	0	0
HSFY1	0	1	0	0
Hsf3	0	1	0	0
IKZF2	0	1	0	0
IKZF4	0	1	0	0
INSM2	0	1	0	0
IRF9	0	1	0	0
IRX4	0	1	0	0
IRX5	0	1	0	0
ISL2	0	1	0	0
KLF14	0	1	0	0
KLF18	0	1	0	0
KLF3	0	1	0	0
LBX2	0	1	0	0

LEUTX	0	1	0	0
LHX6	0	1	0	0
LHX8	0	1	0	0
LHX9	0	1	0	0
LYL1	0	1	0	0
MEIS3P1	0	1	0	0
MEIS3P2	0	1	0	0
MYCL	0	1	0	0
MYCLP1	0	1	0	0
MYNN	0	1	0	0
MYRFL	0	1	0	0
Msx3	0	1	0	0
Mycb	0	1	0	0
NACC1	0	1	0	0
NANOGP1	0	1	0	0
NEUROD4	0	1	0	0
NEUROG1	0	1	0	0
NEUROG2	0	1	0	0
NFE2	0	1	0	0
NFXL1	0	1	0	0
NKX1-1	0	1	0	0
NKX1-2	0	1	0	0
NOTO	0	1	0	0
NPAS1	0	1	0	0
NPAS3	0	1	0	0
OLIG1	0	1	0	0
OLIG3	0	1	0	0
OSR1	0	1	0	0
OVOL3	0	1	0	0
PASD1	0	1	0	0
PATZ1	0	1	0	0
PAX9	0	1	0	0
PBX4	0	1	0	0
PEG3	0	1	0	0

PGBD1	0	1	0	0
PKNOX2	0	1	0	0
POU5F2	0	1	0	0
POU6F2	0	1	0	0
PRDM15	0	1	0	0
PROX2	0	1	0	0
PURG	0	1	0	0
RBAK	0	1	0	0
RELB	0	1	0	0
RFX1	0	1	0	0
RFX7	0	1	0	0
RFX8	0	1	0	0
RHOXF1	0	1	0	0
RHOXF2	0	1	0	0
RHOXF2B	0	1	0	0
RLF	0	1	0	0
SALL3	0	1	0	0
SALL4	0	1	0	0
SCAND2P	0	1	0	0
SCRT2	0	1	0	0
SHOX2	0	1	0	0
SIM1	0	1	0	0
SIM2	0	1	0	0
SIX6	0	1	0	0
SKOR1	0	1	0	0
SKOR2	0	1	0	0
SMAD9	0	1	0	0
SOHLH2	0	1	0	0
SOX3	0	1	0	0
SP110	0	1	0	0
SP140	0	1	0	0
SP140L	0	1	0	0
SP4	0	1	0	0
SP6	0	1	0	0

SP8	0	1	0	0
SP9	0	1	0	0
SPDEF	0	1	0	0
ST18	0	1	0	0
STAT2	0	1	0	0
STOX1	0	1	0	0
STOX2	0	1	0	0
TAL2	0	1	0	0
TBX10	0	1	0	0
TBX22	0	1	0	0
TBX4	0	1	0	0
TBX6	0	1	0	0
TCF23	0	1	0	0
TCF24	0	1	0	0
TFCP2L1	0	1	0	0
TGIF2	0	1	0	0
TGIF2LX	0	1	0	0
TGIF2LY	0	1	0	0
TLX1	0	1	0	0
TLX3	0	1	0	0
TPRX1	0	1	0	0
TSHZ1	0	1	0	0
TSHZ2	0	1	0	0
TSHZ3	0	1	0	0
ZBTB1	0	1	0	0
ZBTB11	0	1	0	0
ZBTB12	0	1	0	0
ZBTB24	0	1	0	0
ZBTB25	0	1	0	0
ZBTB26	0	1	0	0
ZBTB3	0	1	0	0
ZBTB33	0	1	0	0
ZBTB34	0	1	0	0
ZBTB37	0	1	0	0

ZBTB39	0	1	0	0
ZBTB40	0	1	0	0
ZBTB41	0	1	0	0
ZBTB42	0	1	0	0
ZBTB45	0	1	0	0
ZBTB47	0	1	0	0
ZBTB48	0	1	0	0
ZBTB49	0	1	0	0
ZBTB6	0	1	0	0
ZBTB7C	0	1	0	0
ZFHX2	0	1	0	0
ZFHX4	0	1	0	0
ZFP1	0	1	0	0
ZFP14	0	1	0	0
ZFP2	0	1	0	0
ZFP28	0	1	0	0
ZFP3	0	1	0	0
ZFP30	0	1	0	0
ZFP37	0	1	0	0
ZFP41	0	1	0	0
ZFP57	0	1	0	0
ZFP62	0	1	0	0
ZFP69	0	1	0	0
ZFP69B	0	1	0	0
ZFP82	0	1	0	0
ZFP92	0	1	0	0
ZFY	0	1	0	0
ZHX2	0	1	0	0
ZIC4	0	1	0	0
ZIK1	0	1	0	0
ZIM2	0	1	0	0
ZIM3	0	1	0	0
ZKSCAN1	0	1	0	0
ZKSCAN2	0	1	0	0

ZKSCAN4	0	1	0	0
ZKSCAN5	0	1	0	0
ZKSCAN7	0	1	0	0
ZKSCAN8	0	1	0	0
ZNF10	0	1	0	0
ZNF100	0	1	0	0
ZNF101	0	1	0	0
ZNF107	0	1	0	0
ZNF112	0	1	0	0
ZNF114	0	1	0	0
ZNF117	0	1	0	0
ZNF12	0	1	0	0
ZNF121	0	1	0	0
ZNF124	0	1	0	0
ZNF132	0	1	0	0
ZNF133	0	1	0	0
ZNF134	0	1	0	0
ZNF135	0	1	0	0
ZNF136	0	1	0	0
ZNF138	0	1	0	0
ZNF14	0	1	0	0
ZNF141	0	1	0	0
ZNF142	0	1	0	0
ZNF146	0	1	0	0
ZNF154	0	1	0	0
ZNF155	0	1	0	0
ZNF157	0	1	0	0
ZNF16	0	1	0	0
ZNF160	0	1	0	0
ZNF165	0	1	0	0
ZNF169	0	1	0	0
ZNF17	0	1	0	0
ZNF177	0	1	0	0
ZNF18	0	1	0	0

ZNF180	0	1	0	0
ZNF181	0	1	0	0
ZNF182	0	1	0	0
ZNF184	0	1	0	0
ZNF189	0	1	0	0
ZNF19	0	1	0	0
ZNF195	0	1	0	0
ZNF197	0	1	0	0
ZNF2	0	1	0	0
ZNF20	0	1	0	0
ZNF208	0	1	0	0
ZNF211	0	1	0	0
ZNF212	0	1	0	0
ZNF213	0	1	0	0
ZNF214	0	1	0	0
ZNF215	0	1	0	0
ZNF22	0	1	0	0
ZNF221	0	1	0	0
ZNF222	0	1	0	0
ZNF223	0	1	0	0
ZNF225	0	1	0	0
ZNF226	0	1	0	0
ZNF227	0	1	0	0
ZNF229	0	1	0	0
ZNF23	0	1	0	0
ZNF230	0	1	0	0
ZNF232	0	1	0	0
ZNF233	0	1	0	0
ZNF234	0	1	0	0
ZNF235	0	1	0	0
ZNF236	0	1	0	0
ZNF248	0	1	0	0
ZNF25	0	1	0	0
ZNF250	0	1	0	0

ZNF251	0	1	0	0
ZNF253	0	1	0	0
ZNF254	0	1	0	0
ZNF256	0	1	0	0
ZNF257	0	1	0	0
ZNF26	0	1	0	0
ZNF260	0	1	0	0
ZNF264	0	1	0	0
ZNF266	0	1	0	0
ZNF267	0	1	0	0
ZNF268	0	1	0	0
ZNF273	0	1	0	0
ZNF274	0	1	0	0
ZNF275	0	1	0	0
ZNF28	0	1	0	0
ZNF280A	0	1	0	0
ZNF280B	0	1	0	0
ZNF280C	0	1	0	0
ZNF280D	0	1	0	0
ZNF282	0	1	0	0
ZNF283	0	1	0	0
ZNF284	0	1	0	0
ZNF285	0	1	0	0
ZNF286A	0	1	0	0
ZNF286B	0	1	0	0
ZNF287	0	1	0	0
ZNF292	0	1	0	0
ZNF296	0	1	0	0
ZNF3	0	1	0	0
ZNF30	0	1	0	0
ZNF302	0	1	0	0
ZNF304	0	1	0	0
ZNF311	0	1	0	0
ZNF316	0	1	0	0

ZNF317	0	1	0	0
ZNF319	0	1	0	0
ZNF32	0	1	0	0
ZNF320	0	1	0	0
ZNF324	0	1	0	0
ZNF324B	0	1	0	0
ZNF329	0	1	0	0
ZNF331	0	1	0	0
ZNF333	0	1	0	0
ZNF334	0	1	0	0
ZNF337	0	1	0	0
ZNF33A	0	1	0	0
ZNF33B	0	1	0	0
ZNF34	0	1	0	0
ZNF343	0	1	0	0
ZNF345	0	1	0	0
ZNF347	0	1	0	0
ZNF354C	0	1	0	0
ZNF355P	0	1	0	0
ZNF358	0	1	0	0
ZNF362	0	1	0	0
ZNF366	0	1	0	0
ZNF37A	0	1	0	0
ZNF383	0	1	0	0
ZNF391	0	1	0	0
ZNF394	0	1	0	0
ZNF396	0	1	0	0
ZNF397	0	1	0	0
ZNF398	0	1	0	0
ZNF404	0	1	0	0
ZNF408	0	1	0	0
ZNF41	0	1	0	0
ZNF410	0	1	0	0
ZNF415	0	1	0	0

ZNF416	0	1	0	0
ZNF417	0	1	0	0
ZNF418	0	1	0	0
ZNF419	0	1	0	0
ZNF420	0	1	0	0
ZNF423	0	1	0	0
ZNF425	0	1	0	0
ZNF426	0	1	0	0
ZNF429	0	1	0	0
ZNF43	0	1	0	0
ZNF430	0	1	0	0
ZNF431	0	1	0	0
ZNF432	0	1	0	0
ZNF433	0	1	0	0
ZNF436	0	1	0	0
ZNF439	0	1	0	0
ZNF44	0	1	0	0
ZNF440	0	1	0	0
ZNF441	0	1	0	0
ZNF442	0	1	0	0
ZNF443	0	1	0	0
ZNF444	0	1	0	0
ZNF445	0	1	0	0
ZNF446	0	1	0	0
ZNF449	0	1	0	0
ZNF45	0	1	0	0
ZNF454	0	1	0	0
ZNF460	0	1	0	0
ZNF461	0	1	0	0
ZNF467	0	1	0	0
ZNF468	0	1	0	0
ZNF470	0	1	0	0
ZNF471	0	1	0	0
ZNF473	0	1	0	0

ZNF479	0	1	0	0
ZNF48	0	1	0	0
ZNF480	0	1	0	0
ZNF483	0	1	0	0
ZNF484	0	1	0	0
ZNF485	0	1	0	0
ZNF486	0	1	0	0
ZNF487	0	1	0	0
ZNF490	0	1	0	0
ZNF491	0	1	0	0
ZNF492	0	1	0	0
ZNF493	0	1	0	0
ZNF496	0	1	0	0
ZNF497	0	1	0	0
ZNF500	0	1	0	0
ZNF501	0	1	0	0
ZNF502	0	1	0	0
ZNF506	0	1	0	0
ZNF507	0	1	0	0
ZNF510	0	1	0	0
ZNF513	0	1	0	0
ZNF514	0	1	0	0
ZNF516	0	1	0	0
ZNF517	0	1	0	0
ZNF518A	0	1	0	0
ZNF518B	0	1	0	0
ZNF519	0	1	0	0
ZNF521	0	1	0	0
ZNF524	0	1	0	0
ZNF525	0	1	0	0
ZNF526	0	1	0	0
ZNF527	0	1	0	0
ZNF528	0	1	0	0
ZNF529	0	1	0	0

ZNF530	0	1	0	0
ZNF534	0	1	0	0
ZNF540	0	1	0	0
ZNF542P	0	1	0	0
ZNF543	0	1	0	0
ZNF544	0	1	0	0
ZNF546	0	1	0	0
ZNF547	0	1	0	0
ZNF548	0	1	0	0
ZNF549	0	1	0	0
ZNF550	0	1	0	0
ZNF551	0	1	0	0
ZNF552	0	1	0	0
ZNF554	0	1	0	0
ZNF555	0	1	0	0
ZNF556	0	1	0	0
ZNF557	0	1	0	0
ZNF558	0	1	0	0
ZNF559	0	1	0	0
ZNF560	0	1	0	0
ZNF561	0	1	0	0
ZNF562	0	1	0	0
ZNF563	0	1	0	0
ZNF564	0	1	0	0
ZNF565	0	1	0	0
ZNF566	0	1	0	0
ZNF567	0	1	0	0
ZNF568	0	1	0	0
ZNF569	0	1	0	0
ZNF57	0	1	0	0
ZNF570	0	1	0	0
ZNF571	0	1	0	0
ZNF572	0	1	0	0
ZNF573	0	1	0	0

ZNF574	0	1	0	0
ZNF575	0	1	0	0
ZNF576	0	1	0	0
ZNF577	0	1	0	0
ZNF578	0	1	0	0
ZNF580	0	1	0	0
ZNF581	0	1	0	0
ZNF582	0	1	0	0
ZNF583	0	1	0	0
ZNF584	0	1	0	0
ZNF585A	0	1	0	0
ZNF585B	0	1	0	0
ZNF586	0	1	0	0
ZNF587	0	1	0	0
ZNF587B	0	1	0	0
ZNF594	0	1	0	0
ZNF595	0	1	0	0
ZNF596	0	1	0	0
ZNF597	0	1	0	0
ZNF599	0	1	0	0
ZNF600	0	1	0	0
ZNF605	0	1	0	0
ZNF606	0	1	0	0
ZNF607	0	1	0	0
ZNF610	0	1	0	0
ZNF611	0	1	0	0
ZNF613	0	1	0	0
ZNF614	0	1	0	0
ZNF615	0	1	0	0
ZNF616	0	1	0	0
ZNF619	0	1	0	0
ZNF620	0	1	0	0
ZNF621	0	1	0	0
ZNF623	0	1	0	0

ZNF624	0	1	0	0
ZNF625	0	1	0	0
ZNF626	0	1	0	0
ZNF627	0	1	0	0
ZNF629	0	1	0	0
ZNF630	0	1	0	0
ZNF641	0	1	0	0
ZNF646	0	1	0	0
ZNF648	0	1	0	0
ZNF649	0	1	0	0
ZNF652	0	1	0	0
ZNF654	0	1	0	0
ZNF655	0	1	0	0
ZNF658	0	1	0	0
ZNF658B	0	1	0	0
ZNF66	0	1	0	0
ZNF660	0	1	0	0
ZNF662	0	1	0	0
ZNF664	0	1	0	0
ZNF665	0	1	0	0
ZNF667	0	1	0	0
ZNF669	0	1	0	0
ZNF670	0	1	0	0
ZNF671	0	1	0	0
ZNF672	0	1	0	0
ZNF674	0	1	0	0
ZNF675	0	1	0	0
ZNF676	0	1	0	0
ZNF677	0	1	0	0
ZNF678	0	1	0	0
ZNF679	0	1	0	0
ZNF680	0	1	0	0
ZNF681	0	1	0	0
ZNF682	0	1	0	0

ZNF683	0	1	0	0
ZNF684	0	1	0	0
ZNF688	0	1	0	0
ZNF689	0	1	0	0
ZNF69	0	1	0	0
ZNF691	0	1	0	0
ZNF695	0	1	0	0
ZNF696	0	1	0	0
ZNF697	0	1	0	0
ZNF699	0	1	0	0
ZNF7	0	1	0	0
ZNF70	0	1	0	0
ZNF700	0	1	0	0
ZNF701	0	1	0	0
ZNF704	0	1	0	0
ZNF705A	0	1	0	0
ZNF705B	0	1	0	0
ZNF705D	0	1	0	0
ZNF705E	0	1	0	0
ZNF705G	0	1	0	0
ZNF707	0	1	0	0
ZNF708	0	1	0	0
ZNF709	0	1	0	0
ZNF71	0	1	0	0
ZNF710	0	1	0	0
ZNF711	0	1	0	0
ZNF713	0	1	0	0
ZNF714	0	1	0	0
ZNF716	0	1	0	0
ZNF717	0	1	0	0
ZNF718	0	1	0	0
ZNF721	0	1	0	0
ZNF723	0	1	0	0
ZNF724	0	1	0	0

ZNF726	0	1	0	0
ZNF726P1	0	1	0	0
ZNF727	0	1	0	0
ZNF728	0	1	0	0
ZNF729	0	1	0	0
ZNF730	0	1	0	0
ZNF732	0	1	0	0
ZNF735	0	1	0	0
ZNF736	0	1	0	0
ZNF737	0	1	0	0
ZNF74	0	1	0	0
ZNF740	0	1	0	0
ZNF749	0	1	0	0
ZNF75A	0	1	0	0
ZNF75CP	0	1	0	0
ZNF75D	0	1	0	0
ZNF761	0	1	0	0
ZNF763	0	1	0	0
ZNF764	0	1	0	0
ZNF765	0	1	0	0
ZNF768	0	1	0	0
ZNF77	0	1	0	0
ZNF770	0	1	0	0
ZNF771	0	1	0	0
ZNF772	0	1	0	0
ZNF773	0	1	0	0
ZNF774	0	1	0	0
ZNF775	0	1	0	0
ZNF776	0	1	0	0
ZNF777	0	1	0	0
ZNF778	0	1	0	0
ZNF780A	0	1	0	0
ZNF780B	0	1	0	0
ZNF782	0	1	0	0

ZNF783	0	1	0	0
ZNF784	0	1	0	0
ZNF785	0	1	0	0
ZNF786	0	1	0	0
ZNF787	0	1	0	0
ZNF789	0	1	0	0
ZNF79	0	1	0	0
ZNF790	0	1	0	0
ZNF791	0	1	0	0
ZNF792	0	1	0	0
ZNF793	0	1	0	0
ZNF799	0	1	0	0
ZNF8	0	1	0	0
ZNF80	0	1	0	0
ZNF805	0	1	0	0
ZNF808	0	1	0	0
ZNF81	0	1	0	0
ZNF813	0	1	0	0
ZNF814	0	1	0	0
ZNF816	0	1	0	0
ZNF818P	0	1	0	0
ZNF821	0	1	0	0
ZNF823	0	1	0	0
ZNF826P	0	1	0	0
ZNF829	0	1	0	0
ZNF83	0	1	0	0
ZNF835	0	1	0	0
ZNF836	0	1	0	0
ZNF837	0	1	0	0
ZNF84	0	1	0	0
ZNF840P	0	1	0	0
ZNF841	0	1	0	0
ZNF844	0	1	0	0
ZNF845	0	1	0	0

ZNF846	0	1	0	0
ZNF850	0	1	0	0
ZNF852	0	1	0	0
ZNF853	0	1	0	0
ZNF860	0	1	0	0
ZNF865	0	1	0	0
ZNF875	0	1	0	0
ZNF876P	0	1	0	0
ZNF878	0	1	0	0
ZNF879	0	1	0	0
ZNF880	0	1	0	0
ZNF883	0	1	0	0
ZNF888	0	1	0	0
ZNF891	0	1	0	0
ZNF90	0	1	0	0
ZNF92	0	1	0	0
ZNF98	0	1	0	0
ZNF99	0	1	0	0
ZSCAN1	0	1	0	0
ZSCAN12	0	1	0	0
ZSCAN16	0	1	0	0
ZSCAN18	0	1	0	0
ZSCAN2	0	1	0	0
ZSCAN20	0	1	0	0
ZSCAN22	0	1	0	0
ZSCAN23	0	1	0	0
ZSCAN25	0	1	0	0
ZSCAN26	0	1	0	0
ZSCAN29	0	1	0	0
ZSCAN30	0	1	0	0
ZSCAN31	0	1	0	0
ZSCAN32	0	1	0	0
ZSCAN4	0	1	0	0
ZSCAN5A	0	1	0	0

ZSCAN5B	0	1	0	0
ZSCAN5C	0	1	0	0
ZSCAN5DP	0	1	0	0
ZSCAN9	0	1	0	0
Zfp11	0	1	0	0
Zfp120	0	1	0	0
Zfp54	0	1	0	0
Zfp58	0	1	0	0
Zfp809	0	1	0	0
Zfy1	0	1	0	0
Zfy2	0	1	0	0
Znf239	0	1	0	0
Znf271	0	1	0	0
Znf354c	0	1	0	0
Znf431	0	1	0	0
Znf568	0	1	0	0
Znf667	0	1	0	0
Znf728	0	1	0	0
Znf768	0	1	0	0
ADNP2	0	0	1	0
Arap1	0	0	1	0
CREBL2	0	0	1	0
CREBZF	0	0	1	0
DBX1	0	0	1	0
DBX2	0	0	1	0
FOXG1	0	0	1	0
FOXR2	0	0	1	0
HLX	0	0	1	0
HMGA1	0	0	1	0
HOXD12	0	0	1	0
JPH2	0	0	1	0
MNX1	0	0	1	0
P2rx2	0	0	1	0
PREB	0	0	1	0

Rpf2	0	0	1	0
SS18L1	0	0	1	0
TCERG1	0	0	1	0
UNCX	0	0	1	0
VSX1	0	0	1	0
ZBED3	0	0	1	0
ZBTB22	0	0	1	0
ZBTB43	0	0	1	0
ZBTB8B	0	0	1	0
FOXO3B	0	0	0	1
HOXC12	0	0	0	1
NFILZ	0	0	0	1
Rhox13	0	0	0	1
TSTD1	0	0	0	1

GO-coTF (as of 30.05.2023)

Protein	Experimental evidence	Phylogenetic evidence	Manual computational evidence	Automatic computational evidence
ABL1	1	0	0	0
ABT1	1	0	0	0
ACTL6A	1	0	0	0
ACTL6B	1	0	0	0
ACTN1	1	0	0	0
ACTN2	1	0	0	0
ACTN4	1	0	0	0
AEBP2	1	0	0	0
AIM2	1	0	0	0
AIP	1	0	0	0
AJUBA	1	0	0	0
ANKRD1	1	0	0	0
APBB1	1	0	0	0
APEX1	1	0	0	0
ARID1A	1	0	0	0
ARID1B	1	0	0	0
ARID3A	1	0	0	0
ARID5A	1	0	0	0

ARID5B	1	0	0	0
ARL2BP	1	0	0	0
ARRB1	1	0	0	0
ASXL1	1	0	0	0
ATF7IP	1	0	0	0
ATN1	1	0	0	0
ATXN7L3	1	0	0	0
BASP1	1	0	0	0
BCL10	1	0	0	0
BCL11A	1	0	0	0
BCL11B	1	0	0	0
BCL3	1	0	0	0
BCL9L	1	0	0	0
BCOR	1	0	0	0
BEND6	1	0	0	0
BHLHE41	1	0	0	0
BIRC2	1	0	0	0
BRCA1	1	0	0	0
BRD4	1	0	0	0
BRD7	1	0	0	0
BRD8	1	0	0	0
BRDT	1	0	0	0
BTAF1	1	0	0	0
BTG1	1	0	0	0
BTG2	1	0	0	0
BUD31	1	0	0	0
C1D	1	0	0	0
C1QBP	1	0	0	0
CALCOCO1	1	0	0	0
CAMTA2	1	0	0	0
CARM1	1	0	0	0
CASP8AP2	1	0	0	0
CBFA2T2	1	0	0	0
CBX4	1	0	0	0

CCAR1	1	0	0	0
CCDC62	1	0	0	0
CCND1	1	0	0	0
CDYL	1	0	0	0
CEBPZ	1	0	0	0
CENPJ	1	0	0	0
CIITA	1	0	0	0
CIR1	1	0	0	0
CITED1	1	0	0	0
CITED2	1	0	0	0
CITED4	1	0	0	0
CNOT2	1	0	0	0
CNOT6	1	0	0	0
CNOT7	1	0	0	0
CNOT9	1	0	0	0
COPS2	1	0	0	0
COPS5	1	0	0	0
CREBBP	1	0	0	0
CREG1	1	0	0	0
CRYM	1	0	0	0
CTBP1	1	0	0	0
CTBP2	1	0	0	0
CTNNB1	1	0	0	0
Cmtm2a	1	0	0	0
Cys1	1	0	0	0
DAXX	1	0	0	0
DCAF6	1	0	0	0
DCC	1	0	0	0
DDX1	1	0	0	0
DDX17	1	0	0	0
DDX5	1	0	0	0
DDX54	1	0	0	0
DHRS7B	1	0	0	0
DHX9	1	0	0	0

DMAP1	1	0	0	0
DNAJB1	1	0	0	0
DNMT3A	1	0	0	0
DNMT3B	1	0	0	0
DOT1L	1	0	0	0
DRAP1	1	0	0	0
DTX1	1	0	0	0
DYRK1B	1	0	0	0
EDF1	1	0	0	0
EID1	1	0	0	0
ELANE	1	0	0	0
ELK1	1	0	0	0
ENO1	1	0	0	0
ENY2	1	0	0	0
EP300	1	0	0	0
ETS1	1	0	0	0
EZH1	1	0	0	0
EZH2	1	0	0	0
Elob	1	0	0	0
FGF2	1	0	0	0
FHL2	1	0	0	0
FHL5	1	0	0	0
FLYWCH1	1	0	0	0
FOXP3	1	0	0	0
FUS	1	0	0	0
GMNN	1	0	0	0
GON4L	1	0	0	0
GPS2	1	0	0	0
GTF2A1L	1	0	0	0
HCFC1	1	0	0	0
HDAC1	1	0	0	0
HDAC3	1	0	0	0
HDAC4	1	0	0	0
HDAC5	1	0	0	0

HDAC7	1	0	0	0
HDAC9	1	0	0	0
HDGF	1	0	0	0
HELZ2	1	0	0	0
HIPK2	1	0	0	0
HIRA	1	0	0	0
HMGA1	1	0	0	0
HMGA2	1	0	0	0
HMGB1	1	0	0	0
HMGB2	1	0	0	0
HNRNPU	1	0	0	0
HR	1	0	0	0
HSBP1	1	0	0	0
HSPA1A	1	0	0	0
HTATIP2	1	0	0	0
HYAL2	1	0	0	0
IFI16	1	0	0	0
IL31RA	1	0	0	0
ING4	1	0	0	0
IRF2BP1	1	0	0	0
IRF2BP2	1	0	0	0
IRF4	1	0	0	0
JADE1	1	0	0	0
JAZF1	1	0	0	0
JMY	1	0	0	0
JUP	1	0	0	0
KAT2A	1	0	0	0
KAT2B	1	0	0	0
KAT5	1	0	0	0
KAT6A	1	0	0	0
KAT6B	1	0	0	0
KAT8	1	0	0	0
KCTD1	1	0	0	0
KDM1A	1	0	0	0

KDM3A	1	0	0	0
KDM5A	1	0	0	0
KDM5B	1	0	0	0
KMT2E	1	0	0	0
KMT5A	1	0	0	0
LCOR	1	0	0	0
LDB1	1	0	0	0
LDB2	1	0	0	0
LIMD1	1	0	0	0
LMCD1	1	0	0	0
LMO2	1	0	0	0
LMO4	1	0	0	0
LPIN1	1	0	0	0
LPIN2	1	0	0	0
LPXN	1	0	0	0
MAGED1	1	0	0	0
MAK	1	0	0	0
MAML1	1	0	0	0
MAML2	1	0	0	0
MAML3	1	0	0	0
MAP3K10	1	0	0	0
MECP2	1	0	0	0
MED1	1	0	0	0
MED12	1	0	0	0
MED13	1	0	0	0
MED14	1	0	0	0
MED16	1	0	0	0
MED17	1	0	0	0
MED21	1	0	0	0
MED23	1	0	0	0
MED24	1	0	0	0
MED26	1	0	0	0
MED27	1	0	0	0
MED30	1	0	0	0

MED4	1	0	0	0
MED6	1	0	0	0
MED7	1	0	0	0
MID2	1	0	0	0
MLIP	1	0	0	0
MMS19	1	0	0	0
MNDA	1	0	0	0
MRTFA	1	0	0	0
MRTFB	1	0	0	0
MTA1	1	0	0	0
MTA2	1	0	0	0
MTDH	1	0	0	0
MUC1	1	0	0	0
MYBBP1A	1	0	0	0
MYCBP	1	0	0	0
MYOCD	1	0	0	0
MYSM1	1	0	0	0
MYT1L	1	0	0	0
N4BP2L2	1	0	0	0
NACA	1	0	0	0
NCOA1	1	0	0	0
NCOA2	1	0	0	0
NCOA3	1	0	0	0
NCOA4	1	0	0	0
NCOA5	1	0	0	0
NCOA6	1	0	0	0
NCOA7	1	0	0	0
NCOR1	1	0	0	0
NCOR2	1	0	0	0
NFE4	1	0	0	0
NFKBIB	1	0	0	0
NFKBIE	1	0	0	0
NIBAN2	1	0	0	0
NKX2-1	1	0	0	0

NKX2-4	1	0	0	0
NME2	1	0	0	0
NOC2L	1	0	0	0
NOTCH1	1	0	0	0
NPAT	1	0	0	0
NPM1	1	0	0	0
NR0B1	1	0	0	0
NR0B2	1	0	0	0
NR3C1	1	0	0	0
NRG1	1	0	0	0
NRIP1	1	0	0	0
NSD1	1	0	0	0
NUCKS1	1	0	0	0
NUP98	1	0	0	0
NUPR1	1	0	0	0
PA2G4	1	0	0	0
PAGE4	1	0	0	0
PARK7	1	0	0	0
PARP15	1	0	0	0
PARP9	1	0	0	0
PAWR	1	0	0	0
PBXIP1	1	0	0	0
PCBD1	1	0	0	0
PDLIM1	1	0	0	0
PER2	1	0	0	0
PEX14	1	0	0	0
PFDN5	1	0	0	0
PHB	1	0	0	0
PHF12	1	0	0	0
PHF2	1	0	0	0
PIAS4	1	0	0	0
PIR	1	0	0	0
PKM	1	0	0	0
PKN1	1	0	0	0

PMF1	1	0	0	0
PML	1	0	0	0
POU2AF1	1	0	0	0
PPARG	1	0	0	0
PPARGC1A	1	0	0	0
PPARGC1B	1	0	0	0
PPP1R13L	1	0	0	0
PQBP1	1	0	0	0
PRDM16	1	0	0	0
PRDM8	1	0	0	0
PRKCB	1	0	0	0
PRKN	1	0	0	0
PRMT2	1	0	0	0
PRMT5	1	0	0	0
PSIP1	1	0	0	0
PSMC3IP	1	0	0	0
PSMD9	1	0	0	0
PTPN14	1	0	0	0
PYHIN1	1	0	0	0
Pus1	1	0	0	0
RALY	1	0	0	0
RAP2C	1	0	0	0
RB1	1	0	0	0
RBBP8	1	0	0	0
RBCK1	1	0	0	0
RBFOX2	1	0	0	0
RBM14	1	0	0	0
RBPMS	1	0	0	0
RCOR1	1	0	0	0
RCOR2	1	0	0	0
RERE	1	0	0	0
RIOX2	1	0	0	0
RIPK3	1	0	0	0
RLIM	1	0	0	0

RNF14	1	0	0	0
RNF20	1	0	0	0
RNF4	1	0	0	0
RRP1B	1	0	0	0
RUNX1T1	1	0	0	0
RUVBL1	1	0	0	0
RUVBL2	1	0	0	0
RXRB	1	0	0	0
RYBP	1	0	0	0
Rad54l2	1	0	0	0
SAP30	1	0	0	0
SCAI	1	0	0	0
SDR16C5	1	0	0	0
SERTAD2	1	0	0	0
SF1	1	0	0	0
SFMBT1	1	0	0	0
SFR1	1	0	0	0
SIAH2	1	0	0	0
SIN3A	1	0	0	0
SIN3B	1	0	0	0
SIRT1	1	0	0	0
SIRT6	1	0	0	0
SKI	1	0	0	0
SLC30A9	1	0	0	0
SMARCA2	1	0	0	0
SMARCA4	1	0	0	0
SMARCB1	1	0	0	0
SMARCC1	1	0	0	0
SMARCC2	1	0	0	0
SMARCD3	1	0	0	0
SMARCE1	1	0	0	0
SMYD1	1	0	0	0
SND1	1	0	0	0
SNW1	1	0	0	0

SPEN	1	0	0	0
SQSTM1	1	0	0	0
SRA1	1	0	0	0
SRCAP	1	0	0	0
SRSF2	1	0	0	0
SS18	1	0	0	0
SSBP2	1	0	0	0
SSBP3	1	0	0	0
SSBP4	1	0	0	0
SSX1	1	0	0	0
SUB1	1	0	0	0
SUFU	1	0	0	0
SUPT3H	1	0	0	0
SUPT7L	1	0	0	0
TACC1	1	0	0	0
TADA1	1	0	0	0
TADA3	1	0	0	0
TAF11	1	0	0	0
TAF12	1	0	0	0
TAF5L	1	0	0	0
TAF6L	1	0	0	0
TAF9	1	0	0	0
TAF9B	1	0	0	0
TBL1X	1	0	0	0
TBL1XR1	1	0	0	0
TBL1Y	1	0	0	0
TCERG1	1	0	0	0
TCF20	1	0	0	0
TCF25	1	0	0	0
TCF4	1	0	0	0
TCP10L	1	0	0	0
TDG	1	0	0	0
TDP2	1	0	0	0
TDRD3	1	0	0	0

TFAP2A	1	0	0	0
TGFB1I1	1	0	0	0
THRAP3	1	0	0	0
TLE1	1	0	0	0
TLE2	1	0	0	0
TLE4	1	0	0	0
TLE5	1	0	0	0
TMF1	1	0	0	0
TOB1	1	0	0	0
TOX2	1	0	0	0
TOX3	1	0	0	0
TP53BP1	1	0	0	0
TRERF1	1	0	0	0
TRIB3	1	0	0	0
TRIM13	1	0	0	0
TRIM14	1	0	0	0
TRIM15	1	0	0	0
TRIM21	1	0	0	0
TRIM22	1	0	0	0
TRIM24	1	0	0	0
TRIM25	1	0	0	0
TRIM27	1	0	0	0
TRIM28	1	0	0	0
TRIM31	1	0	0	0
TRIM32	1	0	0	0
TRIM37	1	0	0	0
TRIM38	1	0	0	0
TRIM5	1	0	0	0
TRIM52	1	0	0	0
TRIM62	1	0	0	0
TRIM8	1	0	0	0
TRIP11	1	0	0	0
TRIP13	1	0	0	0
TRIP4	1	0	0	0

TRRAP	1	0	0	0
TSG101	1	0	0	0
UBE2L3	1	0	0	0
UBE3A	1	0	0	0
URI1	1	0	0	0
USP16	1	0	0	0
USP21	1	0	0	0
USP22	1	0	0	0
UTF1	1	0	0	0
UXT	1	0	0	0
VGLL1	1	0	0	0
VGLL2	1	0	0	0
VHL	1	0	0	0
WBP2	1	0	0	0
WDR77	1	0	0	0
WNT3A	1	0	0	0
WNT4	1	0	0	0
WWC1	1	0	0	0
WWOX	1	0	0	0
WWTR1	1	0	0	0
Wtip	1	0	0	0
XPC	1	0	0	0
YAF2	1	0	0	0
YAP1	1	0	0	0
ZBED1	1	0	0	0
ZBTB32	1	0	0	0
ZCCHC12	1	0	0	0
ZCCHC18	1	0	0	0
ZFPM1	1	0	0	0
ZFPM2	1	0	0	0
ZIC1	1	0	0	0
ZIC2	1	0	0	0
ZIC3	1	0	0	0
ZMIZ1	1	0	0	0

ZMIZ2	1	0	0	0
ZMYND11	1	0	0	0
ZMYND8	1	0	0	0
ZNF366	1	0	0	0
ZNF451	1	0	0	0
ZNF653	1	0	0	0
ZXDA	1	0	0	0
ZXDC	1	0	0	0
AKIRIN1	0	1	0	0
AKIRIN2	0	1	0	0
ATF7IP2	0	1	0	0
BCLAF1	0	1	0	0
BCLAF3	0	1	0	0
Basp1	0	1	0	0
CAMTA1	0	1	0	0
CBFA2T3	0	1	0	0
CBFB	0	1	0	0
CCAR2	0	1	0	0
CCDC124	0	1	0	0
CDY1	0	1	0	0
CDY2A	0	1	0	0
CDYL2	0	1	0	0
DPF1	0	1	0	0
DPF2	0	1	0	0
DPF3	0	1	0	0
DYRK1A	0	1	0	0
EID2	0	1	0	0
EID2B	0	1	0	0
EWSR1	0	1	0	0
FBXL19	0	1	0	0
FHL3	0	1	0	0
HCFC2	0	1	0	0
HDGFL1	0	1	0	0
HDGFL2	0	1	0	0

HDGFL3	0	1	0	0
Hdgfl3	0	1	0	0
IRF2BPL	0	1	0	0
JMJD1C	0	1	0	0
KAT7	0	1	0	0
KDM2A	0	1	0	0
KDM2B	0	1	0	0
KDM3B	0	1	0	0
KDM7A	0	1	0	0
KMT2C	0	1	0	0
KMT2D	0	1	0	0
LMO1	0	1	0	0
LMO3	0	1	0	0
LPIN3	0	1	0	0
MAMSTR	0	1	0	0
MED10	0	1	0	0
MED12L	0	1	0	0
MED13L	0	1	0	0
MED18	0	1	0	0
MED20	0	1	0	0
MED29	0	1	0	0
MED8	0	1	0	0
MIDEAS	0	1	0	0
MIER1	0	1	0	0
MIER2	0	1	0	0
MIER3	0	1	0	0
MPND	0	1	0	0
MTA3	0	1	0	0
Med29	0	1	0	0
NAB1	0	1	0	0
NAB2	0	1	0	0
NCOR1P1	0	1	0	0
PARP10	0	1	0	0
PARP14	0	1	0	0

PHF10	0	1	0	0
PHF8	0	1	0	0
PIAS1	0	1	0	0
PIAS2	0	1	0	0
PIAS3	0	1	0	0
PPRC1	0	1	0	0
PXN	0	1	0	0
RCOR3	0	1	0	0
RSF1	0	1	0	0
SAP18	0	1	0	0
SAP30L	0	1	0	0
SETD3	0	1	0	0
SIRT7	0	1	0	0
SMARCD1	0	1	0	0
SMARCD2	0	1	0	0
SUPT20H	0	1	0	0
SUPT20HL1	0	1	0	0
SUPT20HL2	0	1	0	0
TADA2A	0	1	0	0
TADA2B	0	1	0	0
TAF15	0	1	0	0
TAF6	0	1	0	0
TCERG1L	0	1	0	0
TLE3	0	1	0	0
TLE6	0	1	0	0
TLE7	0	1	0	0
TOB2	0	1	0	0
WBP2NL	0	1	0	0
WTIP	0	1	0	0
YY1AP1	0	1	0	0
ZNF410	0	1	0	0
ZNF541	0	1	0	0
ZNF710	0	1	0	0
ZXDB	0	1	0	0

AASS	0	0	1	0
CNOT6L	0	0	1	0
FIZ1	0	0	1	0
Fgf2	0	0	1	0
HIPK1	0	0	1	0
HIPK3	0	0	1	0
HSPA1B	0	0	1	0
ING5	0	0	1	0
INSM2	0	0	1	0
NRBF2	0	0	1	0
PRPF6	0	0	1	0
SSX2	0	0	1	0
SSX3	0	0	1	0
SSX4	0	0	1	0
SSX5	0	0	1	0
SSX7	0	0	1	0
TRIM34	0	0	1	0
ZNF335	0	0	1	0
BCL9	0	0	0	1
HSBP1L1	0	0	0	1
MCIDAS	0	0	0	1
MED11	0	0	0	1
MED15	0	0	0	1
MED19	0	0	0	1
MED22	0	0	0	1
MED31	0	0	0	1
MED9	0	0	0	1
NMI	0	0	0	1
SFMBT2	0	0	0	1
SS18L1	0	0	0	1
SS18L2	0	0	0	1
TFB2M	0	0	0	1
**GO-GTF (as of 30.05.2023)**				
**Protein**	**Experimental evidence**	**Phylogenetic evidence**	**Manual computational evidence**	**Automatic computational evidence**

CCNH	1	0	0	0
DR1	1	0	0	0
DRAP1	1	0	0	0
FOXA2	1	0	0	0
GTF2A1	1	0	0	0
GTF2A2	1	0	0	0
GTF2B	1	0	0	0
GTF2E1	1	0	0	0
GTF2E2	1	0	0	0
GTF2F1	1	0	0	0
GTF2F2	1	0	0	0
GTF2H2	1	0	0	0
GTF2H3	1	0	0	0
GTF2H4	1	0	0	0
GTF3A	1	0	0	0
GTF3C1	1	0	0	0
GTF3C2	1	0	0	0
GTF3C3	1	0	0	0
GTF3C4	1	0	0	0
GTF3C5	1	0	0	0
PRRX1	1	0	0	0
PRRX2	1	0	0	0
SNAPC1	1	0	0	0
SNAPC2	1	0	0	0
SNAPC3	1	0	0	0
SNAPC4	1	0	0	0
SNAPC5	1	0	0	0
TAF1	1	0	0	0
TAF10	1	0	0	0
TAF11	1	0	0	0
TAF12	1	0	0	0
TAF13	1	0	0	0
TAF1C	1	0	0	0
TAF3	1	0	0	0

TAF4	1	0	0	0
TAF5	1	0	0	0
TAF6	1	0	0	0
TAF7	1	0	0	0
TAF8	1	0	0	0
TBP	1	0	0	0
TBPL1	1	0	0	0
UBTF	1	0	0	0
BRF1	0	1	0	0
RRN3	0	1	0	0
TAF1L	0	1	0	0
TAF2	0	1	0	0
TAF4B	0	1	0	0
TAF7L	0	1	0	0
TAF9	0	1	0	0
TAF9B	0	1	0	0
TBPL2	0	1	0	0
UBTFL1	0	1	0	0
UBTFL6	0	1	0	0
GTF2H2C	0	0	1	0
TAF6L	0	0	0	1

Table S5

Proteins with dual GO annotations: classified as dbTF/GTF, coTF/GTF and dbTF/coTF according to Gene Ontology (as of 30.05.2023).

dbTF/GTF													
Protein	DBD	GO Catalog	ORFeome	TFCat	Ravazi	Saeed	Lambert	Animal TFDB/dbTF	Animal TFDB/coTF	Tcof-DB/dbTF	Tcof-DB/coTF	JASPAR	TFClass
FOXA2	1	1	1	1	1	1	1	1	0	1	0	1	1
PRRX1	1	1	1	1	1	1	1	1	0	1	0	1	1
PRRX2	1	1	1	1	1	1	1	1	0	1	0	1	1
coTF/GTF													
DRAP1	0	0	1	1	1	0	1	0	1	1	0	0	0
TAF11	0	0	1	1	1	0	0	0	1	0	1	0	0

TAF12	0	0	1	1	1	0	0	0	1	0	0	0	0
TAF6	0	0	1	1	1	0	0	0	1	0	0	0	0
TAF6L	0	0	1	1	1	0	0	0	1	0	1	0	0
TAF9	0	0	1	1	1	0	0	0	1	0	0	0	0
TAF9B	0	0	0	1	0	0	0	0	1	0	1	0	0
dbTF/coTF													
BCL11A	1	1	1	1	1	0	1	1	0	1	0	0	1
BCL11B	1	1	1	1	1	0	1	1	0	1	0	1	1
BHLHE41	1	1	1	1	1	1	1	1	0	1	0	1	1
ELK1	1	1	1	1	1	1	1	1	0	1	0	1	1
ENO1	0	1	1	1	1	1	0	0	1	1	0	0	0
ETS1	1	1	1	1	1	1	1	1	0	1	0	1	1
FOXP3	1	1	1	1	1	1	1	1	0	1	0	1	1
HDAC5	0	0	0	1	0	0	0	0	1	0	1	0	0
HDGF	0	0	0	1	0	0	0	0	1	0	0	0	0
HMGA1	0	0	1	1	1	1	1	1	0	1	0	0	1
INSM2	1	1	1	1	1	1	1	1	0	1	0	0	1
IRF4	1	1	1	1	1	1	1	1	0	1	0	1	1
KMT2D	0	0	0	0	1	0	0	1	1	0	1	0	1
MECP2	1	0	1	1	1	0	1	1	0	1	0	0	0
MRTFA	0	0	0	0	0	0	0	0	1	0	1	0	0
MRTFB	0	0	0	0	0	0	0	0	1	0	1	0	0
MYT1L	1	1	1	1	1	1	1	1	0	1	0	0	1
NCOA3	1	0	1	1	1	1	1	0	1	1	0	0	1
NKX2-1	1	1	1	1	1	1	1	1	0	1	0	1	1
NKX2-4	1	1	1	0	0	1	1	1	0	1	0	1	1
NR3C1	1	1	1	1	1	1	1	1	0	1	0	1	1
PPARG	1	1	1	1	1	1	1	1	0	1	0	1	1
PRDM16	1	1	1	1	1	0	1	1	0	1	0	0	1
RXRB	1	1	1	1	1	1	1	1	0	1	0	1	1
SKI	0	1	0	1	0	1	1	0	1	1	0	0	0
SPEN	0	0	1	1	1	0	1	0	1	0	0	0	0

SS18L1	0	0	0	1	0	0	0	0	1	0	1	0	0
TCERG1	0	0	1	1	1	0	0	0	1	0	1	0	0
TCF20	0	0	1	1	1	1	1	0	1	1	0	0	0
TCF4	1	1	1	1	1	1	1	1	0	1	0	1	1
TFAP2A	1	1	1	1	1	1	1	1	0	1	0	1	1
ZBED1	1	1	0	0	0	1	1	1	0	1	0	1	1
ZBTB32	1	1	1	1	1	1	1	1	0	1	0	1	1
ZIC1	1	1	1	1	1	1	1	1	0	1	0	1	1
ZIC2	1	1	1	1	1	1	1	1	0	1	0	1	1
ZIC3	1	1	1	1	1	1	1	1	0	1	0	1	1
ZNF366	1	1	1	1	1	1	1	1	0	1	0	0	1
ZNF410	1	0	1	1	1	1	1	1	0	1	0	1	1
ZNF710	1	1	0	0	0	1	1	1	0	1	0	0	1
